# A green protocol ball milling synthesis of dihydropyrano[2,3-*c*]pyrazole using nano-silica/aminoethylpiperazine as a metal-free catalyst

**DOI:** 10.1186/s13065-023-00934-1

**Published:** 2023-03-04

**Authors:** Dina Mallah, Bi Bi Fatemeh Mirjalili

**Affiliations:** Department of Chemistry, College of Science, Yazd University, P.O. Box 89195-741, Yazd, Islamic Republic of Iran

**Keywords:** Green chemistry, Ball milling, Pyranopyrazoles, Nano-silica/aminoethylpiperazine

## Abstract

**Background:**

Ball mill is an effective, and green method for the synthesis of heterocyclic compounds in very good yields. This method is a simple, economical, and environmentally friendly process. In this work, an efficient approach for the synthesis of pyranopyrazoles (PPzs) using ball milling and metal-free nano-catalyst (Nano-silica/aminoethylpiperazine), under solvent-free conditions was reported.

**Results:**

The new nano-catalyst silica/aminoethylpiperazine was prepared by immobilization of 1-(2-aminoethyl)piperazine on nano-silica chloride. The structure of the prepared nano-catalyst was identified by FT-IR, FESEM, TGA, EDX, EDS-map, XRD, and pH techniques. This novel nano-catalyst was used for the synthesis of dihydropyrano[2,3-*c*]pyrazole derivatives under ball milling and solvent-free conditions.

**Conclusions:**

Unlike other pyranopyrazoles synthesis reactions, this method has advantages including short reaction time (5–20 min), room temperature, and relatively high efficiency, which makes this protocol very attractive for the synthesis of pyranopyrazoles derivatives.

**Supplementary Information:**

The online version contains supplementary material available at 10.1186/s13065-023-00934-1.

## Introduction

Recent advances in chemistry have led to its increasing use of it in various fields of life and industry. In addition to this comfort and well-being, unfortunately, the excessive use of toxic and dangerous substances has caused serious and irreparable damage to the environment and humans. Since the relationship between chemical knowledge and human life is a two-way relationship, the appropriate solution to reduce the risks along with enjoying the benefits of chemistry is the use of green chemistry [1]. Environmental protection has become a very important and popular topic for organic chemists in recent decades. That's why scientists are designing reactions by following green chemistry. Except for green chemistry methods, many of the methods reported for the synthesis of the heterocyclic compound often need hard experimental conditions or have poor yields and by-products [2].

The ball milling method is new and environmentally friendly and is a mechanicochemical method that has recently become very popular and is considered by organic chemists for the synthesis of various organic materials [3]. The advantages of the mechanicochemical methods are that the reactions can be performed under solvent-free conditions, in a short time, and with pure products [4]. In addition, ball milling methods have different applications in medicinal and pharmaceutical chemistry [5, 6], it is also widely used in the polymerization reaction by mediating ball milling [7, 8], multi-component organic synthesis [9–11], including gas reagents [12], carbon materials [13–15], and the preparation of crystals [16, 17].

Pyranopyrazole (PPz) is a fused heterocyclic framework comprising pyran and pyrazole moieties [18–21]. PPzs have shown important roles in the field of medicinal and pharmaceutical chemistry [18] including antimicrobial [22], anti-cancer [23], and anti-fungal [24], and many drugs containing sulfaphenazole (antibacterial) [25], celecoxib (anti-inflammatory) [26, 27], rimonabant (antiobesity) [28], and mepiprazole (antidepressant) [29] are derived from pyrazole core [30, 31]. Several catalysts have been applied for the synthesis of PPz such as Nano-AlPO_4_/Ti(IV) [32], nano-eggshell/Ti(IV) [33], Fe_3_O_4_@SiO_2_@(CH_2_)_3_NH@CC@Imidazole@SO_3_H [34], AC-SO_3_H/[Choline-Cl][Urea]_2_ [35], PAN@melamine/Fe_3_O_4_ [36] isonicotinic acid [37], nano‐Fe‐ [phenylsalicylaldiminemethylpyranopyrazole]Cl_2_ [38], CaO@SiO_2_-SO_3_H [39], IRMOF-3/GO/CuFe_2_O_4_ [40], [(EMIM)Ac)] [41], Ru^III^@CMC/Fe_3_O_4_ [42], PS-DABCO [43], Fe_3_O_4_@rGO-NH [44], and NiFe_2_O_4_@SiO_2_-H_14_[NaP_5_W_30_O_110_] [45].

**Fig. 1:**
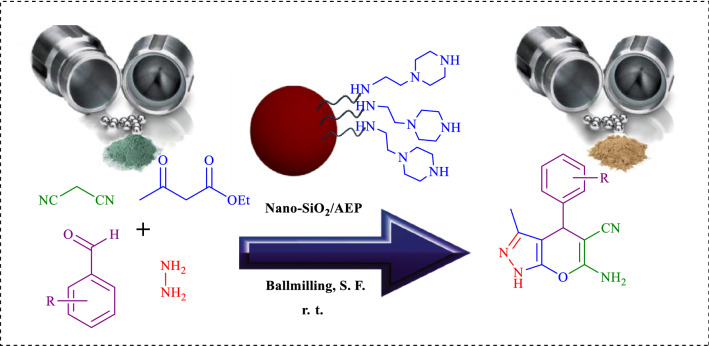
Synthesis of PPz using Nano-SiO_2_/AEP under solvent-free ball milling conditions

In this work, we wish to report an efficient method for the synthesis of PPz using nano-silica/aminoethylpiperazine (Nano-SiO_2_/AEP) under solvent-free ball milling conditions (Fig. 1). The heterogeneous nano-catalyst was identified by the FT-IR, FESEM, TGA, EDX, EDS mapping, XRD, and pH techniques.

## Results and discussion

**Fig. 2:**
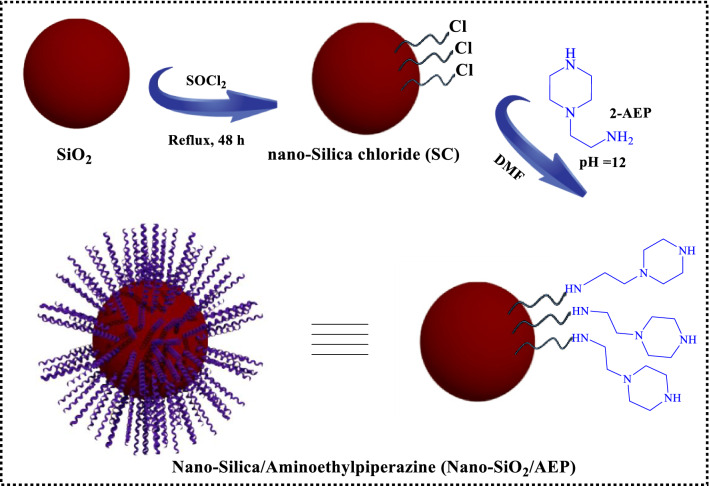
Preparation of Nano-SiO_2_/AEP

To prepare the Nano-SiO_2_/AEP nano-catalyst, first, a mixture of nano-silica gel and thionyl chloride was refluxed for 48 h to prepare the nano-silica chloride. In the next step, the dried silica-chloride was reacted with 2-aminoethylpiperazine (AEP) in DMF at 80 °C (Fig. 2).

### FT-IR of nano-SiO_2_/AEP

**Fig. 3:**
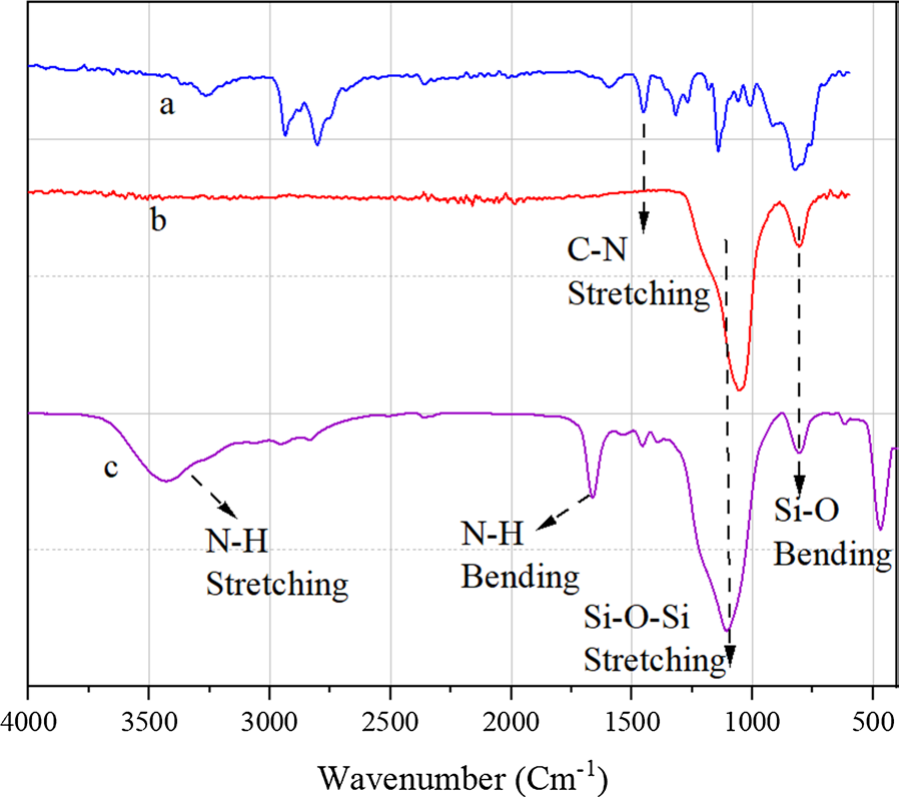
FT-IR of (**a**) 2-AEP, (**b**) nano-silica chloride, (**c**) Nano-SiO_2_/AEP

According to the FT-IR 2-AEP (Fig. 3a), the peaks at 2804 cm^−1^ and 2936 cm^−1^ are due to the symmetric and asymmetric stretching vibration of the CH_2_ group, respectively, and the peak at 1594 cm^−1^ is related to N–H bending vibration. The broad peak at the range of 3200–3400 cm^−1^ is related to N–H stretching vibration. In the spectrum of nano-silica chloride (Fig. 3b), two bands at 806 cm^−1^ and 1055 cm^−1^ are assigned to the stretching and bending vibration of the Si–O–Si, respectively. After immobilization of AEP on nano-silica chloride (Fig. 3c), the peak at 1452 cm^−1^ is assigned to the stretching vibration of the C-N. All of these observations indicate that the Nano-SiO_2_/AEP heterogeneous catalyst has been successfully prepared.

### XRD of nano-SiO_2_/AEP

**Fig. 4:**
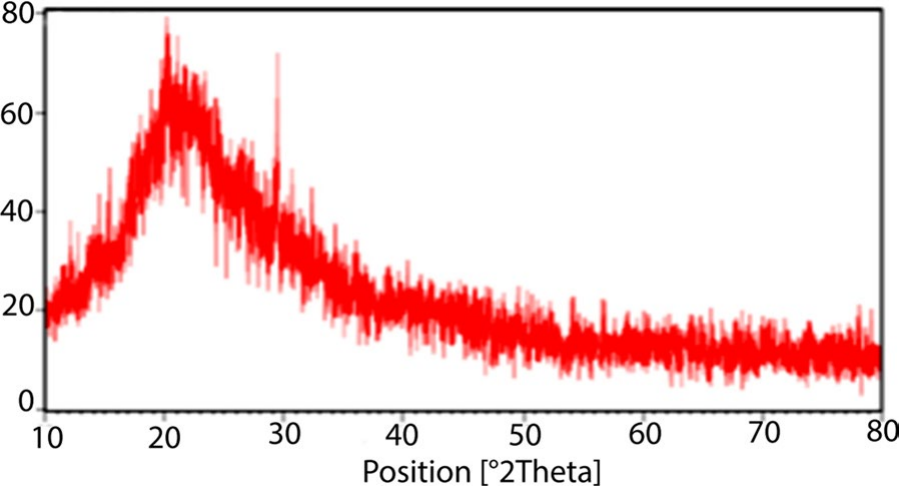
XRD pattern of Nano-SiO_2_/AEP

XRD analysis of the Nano-SiO_2_/AEP spectrum is shown in Fig. 4. A broad peak was observed at 2*θ* = 23–30° which proved that the catalyst is predominantly in the amorphous form. This is in agreement with the literature [46].

### Thermal gravimetric analysis (TGA) of nano-SiO_2_/AEP

**Fig. 5:**
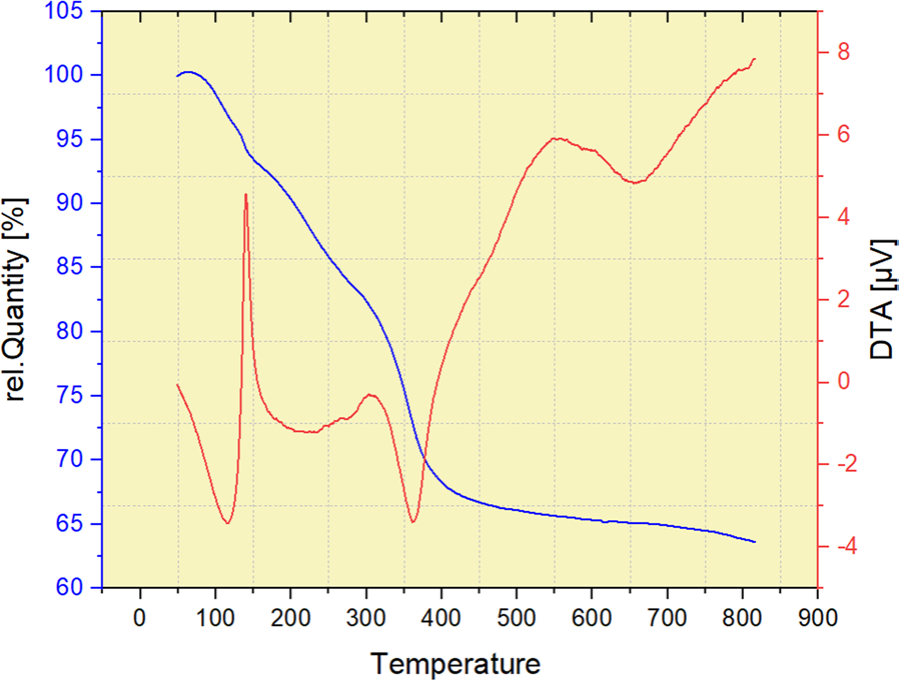
TGA curve of Nano-SiO_2_/AEP

The thermal stability of the Nano-SiO_2_/AEP nano-catalyst was determined by TGA and DTA (Fig. 5) in the temperature range (50–800 °C). The DTA curve shows the endothermic processes in the temperature range of 50–100 °C and 150–350 °C. According to the TGA curve, two stages of weight loss occurred. The first weight loss (less than 100 °C) can be related to the loss of surface water and other solvents on the catalyst surface. The second weight loss of 150–390 °C can be attributed to the destruction of the organic part of the catalyst.

### FESEM of heterogeneous nano-SiO_2_/AEP

**Fig. 6:**
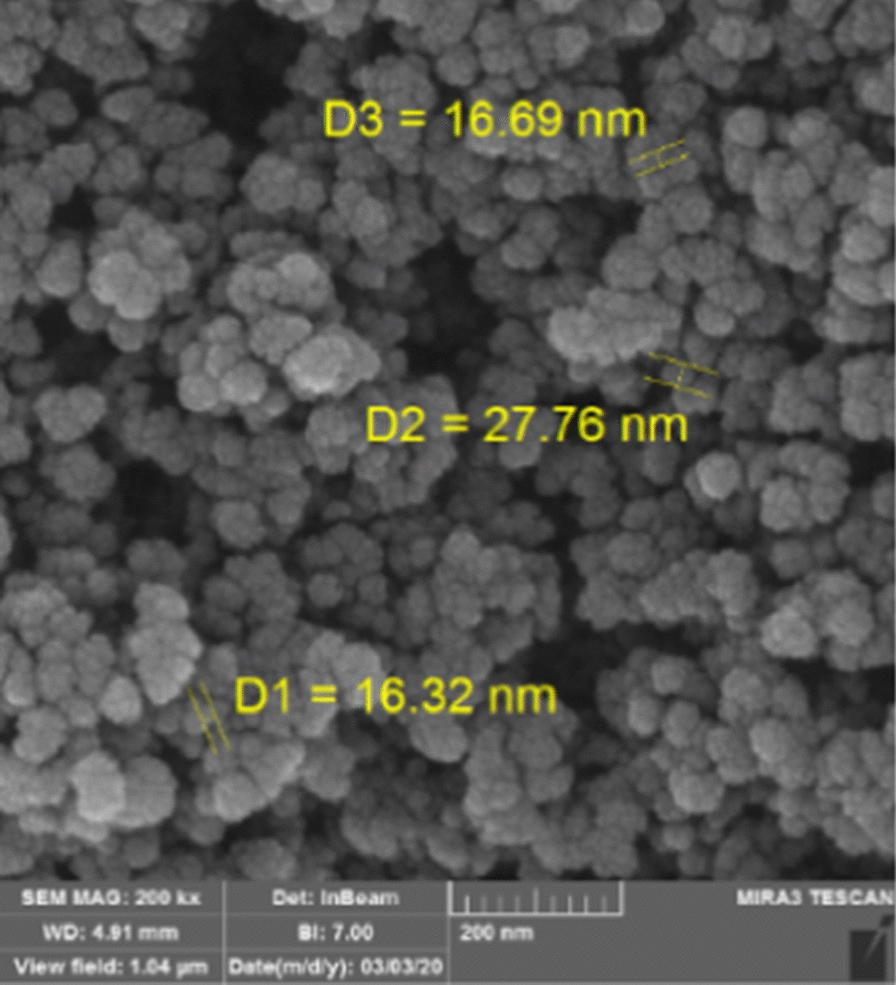
FESEM images of Nano-SiO_2_/AEP

The morphological properties and size of Nano-SiO_2_/AEP nano-catalysts were investigated by FESEM and the results are compiled in Fig. 6. The results show that the Nano-SiO_2_/AEP is composed of a nanoparticle scale that shows a quasi-spherical morphology with diameters in the range of 16–27 nm.

### EDS-map of nano-SiO_2_/AEP and energy dispersive X-ray (EDX)

**Fig. 7:**
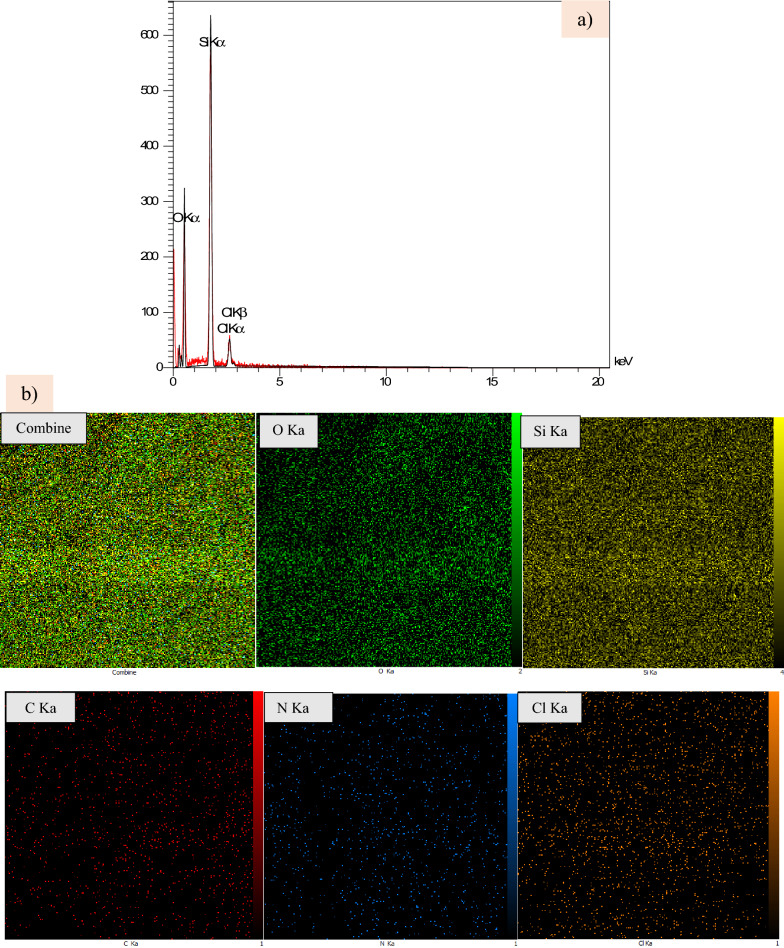
**a** EDX spectrum of Nano-SiO_2_/AEP, **b** Element mapping images for Nano-SiO_2_/AEP nano-catalyst

The EDX spectrum of Nano-SiO_2_/AEP (Fig. 7a), shows the presence of the elements O, Si, C, and N with the corresponding weight percentages (47.70, 21.68, 16.56, 10.99%). The elemental mapping of the Nano-SiO_2_/AEP nano-catalyst is shown in Fig. 7b. According to obtained textures, the elements are homogeneously distributed within the nano-catalyst.

To confirm the basic property of nano-catalyst Nano-SiO_2_/AEP, 0.04 g of it was added to 20 mL of deionized water (pH = 7) and stirred for 1 h at room temperature. Then, the pH of the obtained mixture is 8.62.

**Fig. 8:**
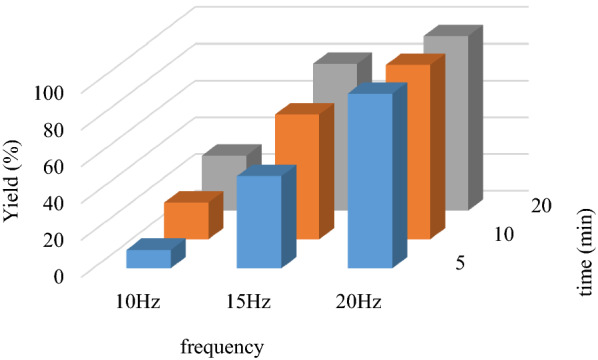
Influence of frequency and milling time on the yield of product

After the characterization of the basic nano-catalyst Nano-SiO_2_/AEP, it was used for the synthesis of pyranopyrazoles. To optimize the reaction conditions, 4-nitrobenzaldehyde, hydrazine hydrate, ethylacetoacetate, and malononitrile were performed under various conditions such as the catalyst amount, temperature, and solvent (Table 1). Meanwhile, the model reaction was done in a stainless steel vial and milled with two stainless steel balls of 0.8 mm diameter at 10, 15, and 20 Hz at room temperature (Fig. 8). At the end of the reaction, hot ethanol was added and the entire reaction mixture was scraped, then the catalyst was separated. When the reaction was performed at lower frequencies such as 10 Hz, a few substrates were still present, probably due to the reduction in the amount of energy per impact.

**Table 1:** Optimization of various reaction conditions^a^


Entry	Conditions	Time (min)	Yield (%)^b^
	Solvent/ Temp. (°C)/ (Catalyst (g))		
1	-/ r. t./ (Nano-SiO_2_/AEP (0.01))	60	49
2	-/ r. t./ (Nano-SiO_2_/AEP (0.02))	60	53
3	-/ r. t./ (Nano-SiO_2_/AEP (0.03))	45	68
4	-/ r. t./ (Nano-SiO_2_/AEP (0.035))	25	87
**5**	**-/ r. t./ (Nano-SiO**_**2**_**/AEP (0.04))**	**5**	**95**
6	-/ r. t./ (Nano-SiO_2_/AEP (0.05))	30	90
7^c^	-/ 80/ (Nano-SiO_2_/AEP (0.04))	80	68
8^c^	-/ 70/ (Nano-SiO_2_/AEP (0.04))	70	73
9^c^	-/ 50/ (Nano-SiO_2_/AEP (0.04))	35	89
10^c^	H_2_O/ r. t./ (Nano-SiO_2_/AEP (0.04))	120	50
11^c^	EtOH/ r. t./ (Nano-SiO_2_/AEP (0.04))	120	49
12^c^	H_2_O/EtOH/r. t./(Nano-SiO_2_/AEP (0.04))	120	70
13^c^	MeOH/ r. t./ (Nano-SiO_2_/AEP (0.04))	120	67
14^c^	CH_3_CN/ r. t./ (Nano-SiO_2_/AEP (0.04))	120	56
15	-/ r. t./ -	120	Trace
16	-/ r.t./ (Nano-SiO_2_ (0.04))	120	Trace
17	-/ r.t./ (Nano-SC (0.04))	120	Trace

Bold values indicate the modified condition for synthesis of pyranopyrazole

^a^Reaction conditions: ethylacetoacetate (1 mmol), hydrazine hydrate (1.5 mmol), malononitrile (1 mmol), and 4-nitrobenzaldehyde (1 mmol) were milled in a 10 mL stainless steel milling vial with two balls at 20 Hz. ^b^Isolated yield. ^c^Normal stirring condition

With the optimal reaction conditions (Table 1, entry 5), the best result was achieved by using a mixer-milling (frequency of 20 Hz) and 0.04 g of catalyst without any solvent.

As Table 2 shows, a wide range of aromatic aldehydes containing electron donors and electron-withdrawing groups were applied for the synthesis of PPz derivatives under optimized conditions.

**Table 2:** Synthesis of PPz derivatives catalyzed by Nano-SiO_2_/AEP at room temperature and solvent-free conditions^a^


Entry	Aldehyde	Time (min)	Yield (%)^b^	m. p. (°C)	
				Found	Reported (ref.)
1	4-Nitrobenzaldehyde	5	95	242–244	246–248 [32]
2	4-Chlorobenzaldehyde	5	93	230–231	235–237 [32]
3	3-Nitrobenzaldehyde	10	76	210–211	235–236 [32]
4	2,4-Dichlorobenzaldehyde	5	87	223–225	223–225 [33]
5	4-Fluorobenzaldehyde	5	96	212–214	212–214 [33]
6	4-Boromobenzaldehyde	5	97	178–180	179–181 [32]
7	3-Methoxy-4-hydroxybenzaldehyde	20	93	233–235	234–236 [33]
8	4-Hydroxybenzaldehyde	10	92	220–221	222–224 [33]
9	4-Methylbenzaldehyde	15	87	204–205	170–173 [32]
10	Benzaldehyde	20	90	241–243	243–244 [35]
11	3,4-Dihydroxybenzaldehyde	15	90	221–224	220–222 [32]
12	Furan-2-carbaldehyde	15	93	234–235	233–235 [32]
13	Pyrrole-2-carbaldehyde	20	94	210–213	-
14	2-Methoxybenzaldehyde	20	80	220–222	214–216 [32]
15	Cyclohexanecarbaldehyde	15	80	138–139	140–142 [32]
16	3-phenylpropionaldehyde	12	90	210–212	–
17	butyraldehyde	20	68	117–118	115–117 [32]

^a^Reaction conditions: ethylacetoacetate (1 mmol), hydrazine hydrate (1.5 mmol), malononitrile (1 mmol), and 4-nitrobenzaldehyde (1 mmol) were milled in a 10 mL stainless steel milling vial with two balls at 20 Hz for 5–20. ^b^Isolated yield

### Reusability of nano-SiO_2_/AEP nano-catalyst

**Fig. 9:**
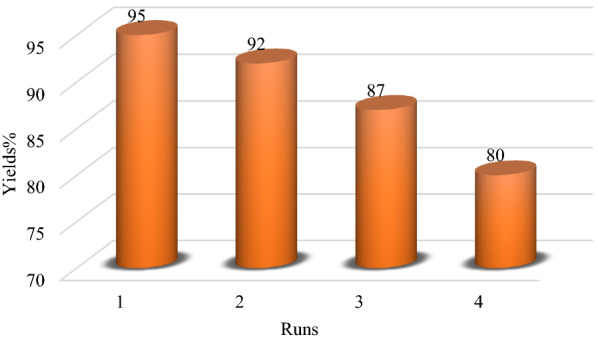
Reusability of Nano-SiO_2_/AEP nano-catalyst

The reusability of nano-catalyst Nano-SiO_2_/AEP was investigated as an important factor for green synthesis (Fig. 9). After completion of the reaction, hot ethanol was added to the reaction mixture and then filtered to separate the nano-catalyst from it. The separated nano-catalyst was washed three times with hot ethanol, dried at ambient temperature, and reused three times with a low loss of its activity.

The catalytic activity of the Nano-SiO_2_/AEP heterogeneous nano-catalyst was compared with other catalysts. As shown in Table 3, Nano-SiO_2_/AEP nano-catalyst acts with relatively high catalytic activity in a short reaction time.

**Table 3:** Comparison of catalytic activity of Nano-SiO_2_/AEP with other catalysts

Entry	Conditions	Time (min)	Yield (%)	References
	Temp. (℃), Solvent, Catalyst			
1	Reflux, EtOH, NAPT	30	82	[46]
2	Reflux, EtOH, Ru^III^@CMC/Fe_3_O_4_	30	93	[42]
3	Reflux, EtOH, PS-DABCO	120	84	[43]
4	r. t., H_2_O, Piperidine	5–10	89	[47]
5	Reflux, EtOH, Et_3_N	15	72	[48]
6	r. t., no solvent, Nano-SiO_2_/AEP	5	95	This work

**Fig. 10:**
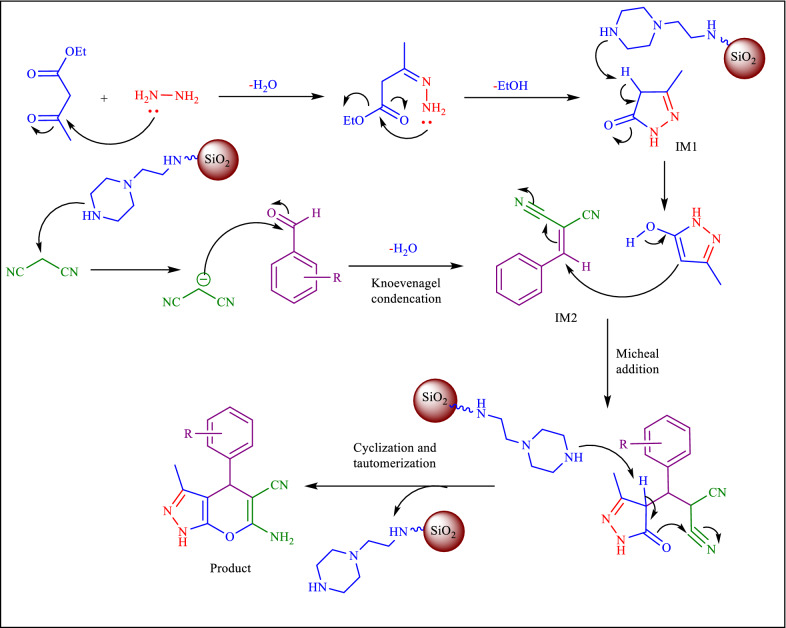
Proposed mechanism for synthesis of PPz in the presence of Nano-SiO_2_/AEP

According to relevant literature reports [18], the proposed mechanism for the synthesis of PPz is shown in Fig. 10. Pyranopyrazole formation is a three-step process. Initially, under ball milling conditions, ethylacetoacetate reacts with hydrazine to form an active pyrazolone intermediate (IM1). Then in the second step, aldehyde with malononitrile is condensed and forms an active intermediate IM2. In the third step, both intermediates, IM1, and IM2 are condensed to produce the desired product.

## Conclusions

In summary, we have developed Nano-SiO_2_/AEP as a new heterogeneous nano-catalyst and identified it by FT-IR, FESEM, TGA, XRD, EDX, EDS-map, and pH techniques. A highly efficient, environmentally friendly practical method for the synthesis of PPz derivatives was developed in the presence of Nano-SiO_2_/AEP nano-catalysts. The reaction proceeds well by ball milling under solvent and metal-free conditions. Other key features of this green approach include high performance, easy preparation, and low temperature.

## Experimental section

### Materials and methods

Chemicals were purchased from Merck, Fluka, and Aldrich Chemical Companies. FT-IR spectra were run on a Bruker, Equinox 55 spectrometer. A Bruker (DRX-400 Avanes) NMR was applied to record the ^1^H-NMR and ^13^C-NMR spectra. Melting points were determined on a Büchi B-540 apparatus. X-ray diffraction (XRD) pattern was obtained by the BRUKER, (Avance). Field Emission Scanning Electron Microscopy (FESEM) images were obtained by a TESCAN, Mira III. The EDS-MAP micrographs were obtained on MIRA II detector SAMX from TESCAN company (France). Thermal gravimetric analysis (TGA) was conducted using the “STA 503” instrument from BAHR company. The pH of the nano-catalyst was matured by the Metrohm model 691 pH/mv meter. The Reactions were conducted using the Mixer Mill model Retsch MM 400 which consisted of two stainless steel vials, each containing two stainless steel balls.

### Preparation of nano-silica chloride (SC)

In a 250 mL round-bottomed flask equipped with a condenser, 10 g of Silica gel and 40 mL of thionyl chloride were added and refluxed for 48 h. Then, the reaction mixture was filtered and the resulting mixture was washed three times with dichloromethane (40 mL × 3) to remove unreacted thionyl chloride from the reaction mixture, the resulting white-grayish powder was dried at ambient temperature and stored in a tight sample tube.

### Preparation of nano-silica chloride/aminoethylpiperazine (nano-SiO_2_/AEP)

In a 100 mL round-bottomed flask, 15 mmol nano-Silica chloride (1.433 g) and 15 mmol of 2-AEP (1.938 g, pH = 12), 10 mL of DMF were heated for 24 h at 80 °C. Then, the resulting mixture was washed three times with dichloromethane (10 mL × 3) and dried at ambient temperature.

### General procedure for the synthesis of PPz

In a stainless steel ball mill vessel, a mixture of ethylacetoacetate (1 mmol), hydrazine hydrate (1.5 mmol), aldehydes (1 mmol), malononitrile (1 mmol), and Nano-SiO_2_/AEP (0.04 g) was milled at 20 Hz. The reaction progress was monitored by TLC (n-hexane: ethyl acetate [8:2]). After checking out the reaction, hot ethanol was added and the reaction mixture was scraped and filtrated to separate the catalyst. Then, the obtained solution was poured into cold water. The crude products appeared as solids that were filtered and washed with water. The crude products were recrystallized in ethanol.

## Supplementary Information

**Additional file 1.** Supplementary file containing FTIR and NMR of products.

## Data Availability

All the methods were carried out in accordance with relevant local/national/international institutional guidelines and regulations. All data generated or analyzed during this study are not publicly available due to DATA NOT PUBLIC but are available from the corresponding author on reasonable request.

## Abbreviations

PPz: Pyranopyrazole
FESEM: Field emission scanning electron microscopy
TGA: Thermo gravimetric analysis
XRD: X-ray diffraction
AEP: 2-Aminoethylpiperazine
DMF: *N*, *N*-Dimethylformamide
IM: Intermediate
