# Supplementary material for: A green protocol ball milling synthesis of dihydropyrano[2,3-c]pyrazole using nano-silica/aminoethylpiperazine as a metal-free catalyst
Source: BMC Chem. 2023 Mar 4;17(1):10. doi: 10.1186/s13065-023-00934-1 (PMC9985283; doi:10.1186/s13065-023-00934-1)
Supplement: Supplementary file 1 — Additional file 1. Supplementary file containing FTIR and NMR of products. [file 13065_2023_934_MOESM1_ESM.docx]

**A green protocol Ball milling synthesis of Dihydropyrano[2,3-*c*]Pyrazole using Nano-Silica/aminoethylpiperazine as a metal-free catalyst**

Dina Mallah, BiBi Fatemeh Mirjalili*

Department of Chemistry, College of Science, Yazd University, P.O. Box 89195-741, Yazd, Iran

*E-mail: fmirjalili@yazd.ac.ir*

***6-Amino-3-methyl-4-(4-nitrophenyl)-1,4-dihydropyrano[2,3-c]pyrazole-5-carbonitrile (Table 2, entry1)***: White solid. m. p. = 242-244 °C. FT- IR (ATR)/ ῡ(cm^-1^):3219, 3103, 2195, 1646, 1593, 1514, 1403, 1351, 1163, 1108, 810, 746.; ^1^H-NMR (400 MHz, Acetone-d_6_)/ δ ppm: 2 (s, 3H), 4.88 (s, 1H), 6.30 ( s, 2H), 7.55 (d, *J* = 8 Hz, 2H), 8.23 (d, *J* = 8 Hz, 2H), 11.43 (s, 1H). ^13^CNMR (100 MHz, DMSO-d_6_)/δ ppm: 161.62, 155.15, 152.59, 146.85, 136.36, 132.19, 129.32, 124.38, 120.98, 97.04, 56.37, 36.36, 10.22.

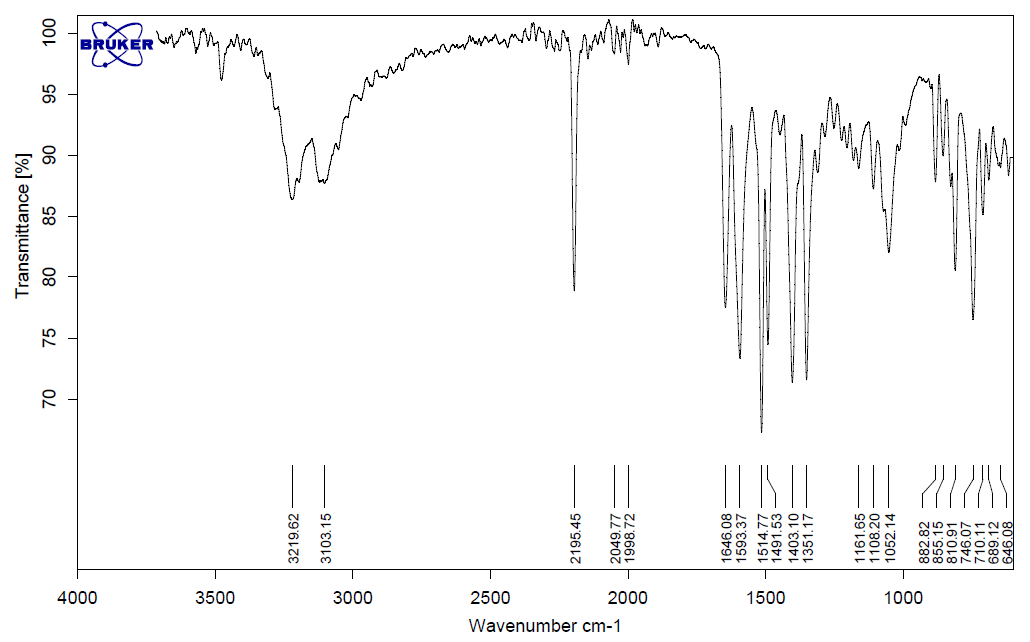


**Fig. S1**. The FT-IR of 6-Amino-3-methyl-4-(4-nitrophenyl)-1,4-dihydropyrano[2,3-*c*]pyrazole-5-carbonitrile


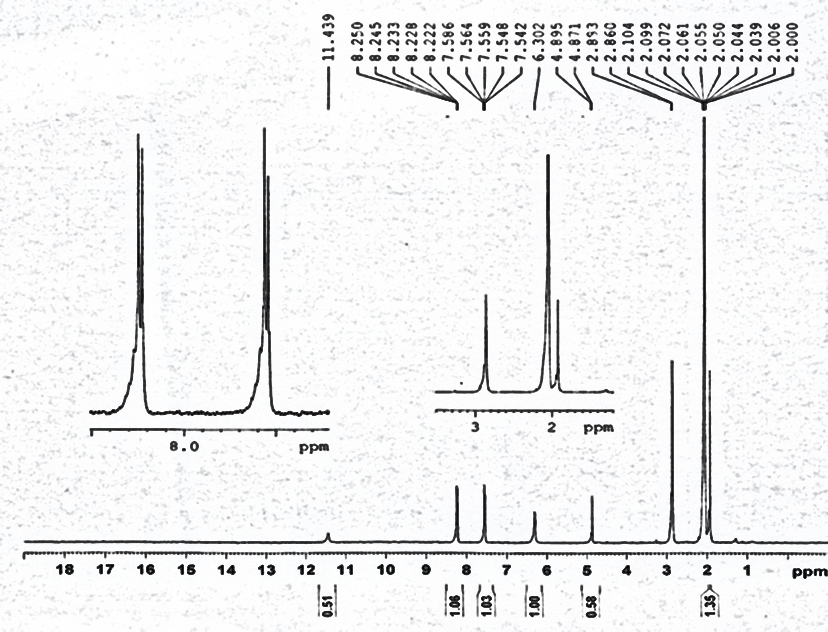


**Fig. S2.** The ^1^H NMR (400 MHz) spectrum of 6-Amino-3-methyl-4-(4-nitrophenyl)-1,4-dihydropyrano[2,3-*c*]pyrazole-5-carbonitrile


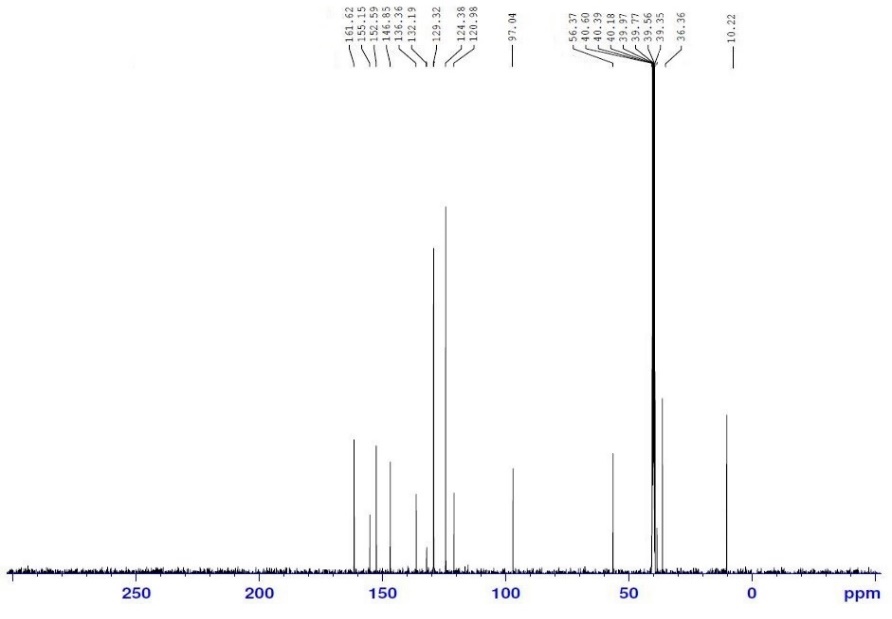


**Fig. S3.** The ^13^C NMR (100 MHz) spectrum of 6-Amino-3-methyl-4-(4-nitrophenyl)-1,4-dihydropyrano[2,3-*c*]pyrazole-5-carbonitrile

***6-Amino-4-(4-chlorophenyl)-3-methyl-1,4-dihydropyrano[2,3-c]pyrazole-5-carbonitrile* *(Table 2, entry 2):*** White solid. m. p. 230-231 °C. FT- IR (ATR) /ῡ (cm^-1)^: 3124, 2192, 1640, 1594, 1489, 1054, 798. ^1^HNMR (400 MHz, DMSO-d_6_)/δ (ppm): 1.81 (s, 3H), 4.65 (s, 1H), 6.96 (s, 2H), 7.21 (d, *J* = 8 Hz, 2H), 7.39 (d, *J* = 8 Hz, 2H), 12.16 (s, 1H).; ^13^C NMR (100 MHz, DMSO-d_6_)/δ ppm: 161.38, 155.18, 143.97, 136.15, 131.71, 130.51, 129.85, 128.94, 121.15, 97.67, 57.19, 36.03, 10.22.

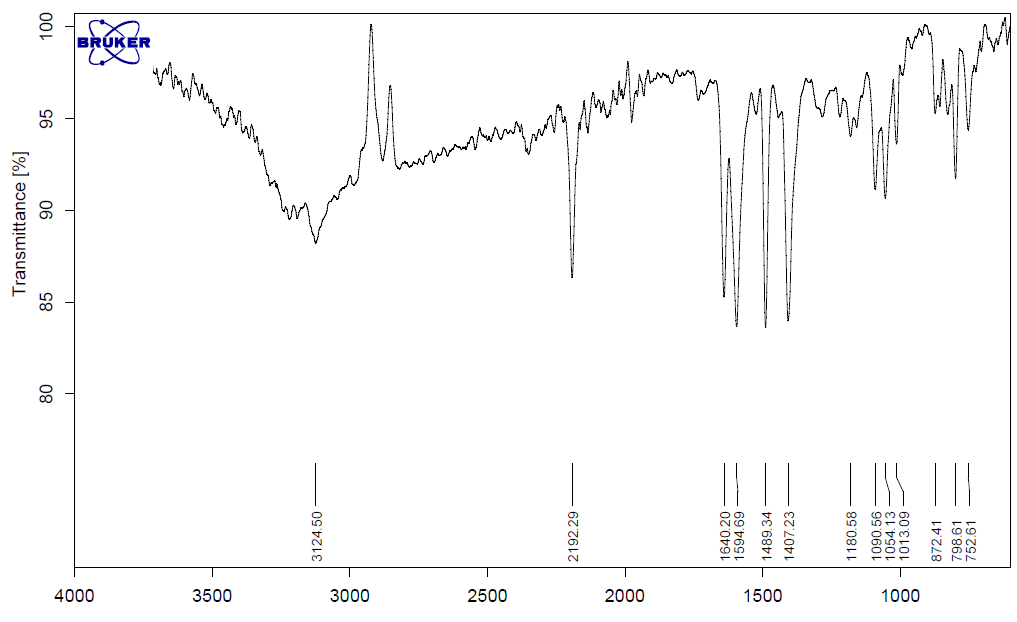


Fig. S4. The FT-IR of 6-Amino-4-(4-chlorophenyl)-3-methyl-1,4-dihydropyrano[2,3-*c*]pyrazole-5-carbonitrile


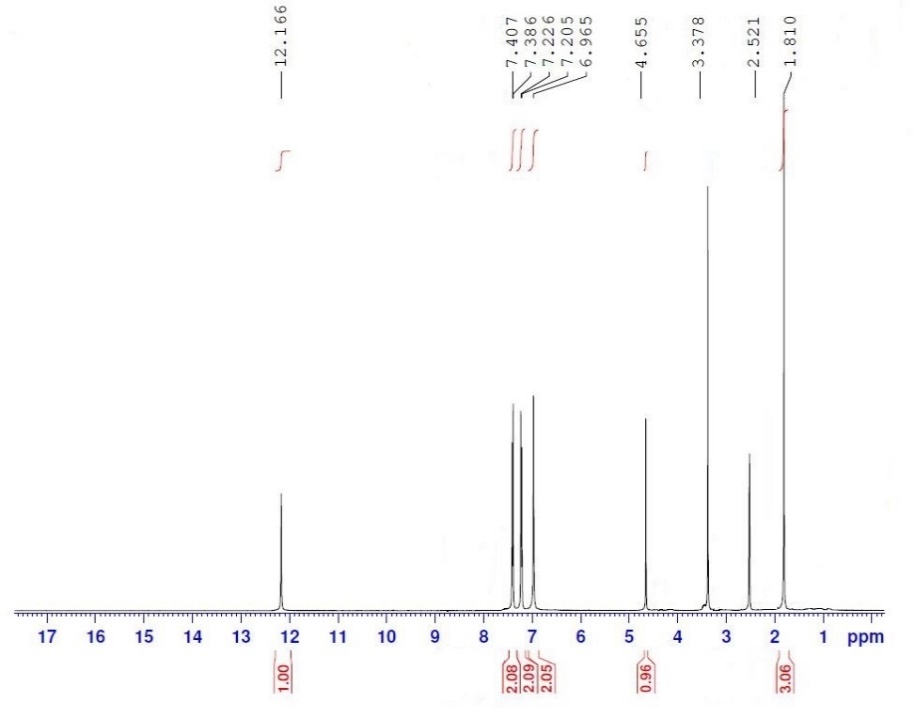


Fig. S5. The ^1^H NMR (400 MHz) spectrum of 6-Amino-4-(4-chlorophenyl)-3-methyl-1,4-dihydropyrano[2,3-*c*]pyrazole-5-carbonitrile


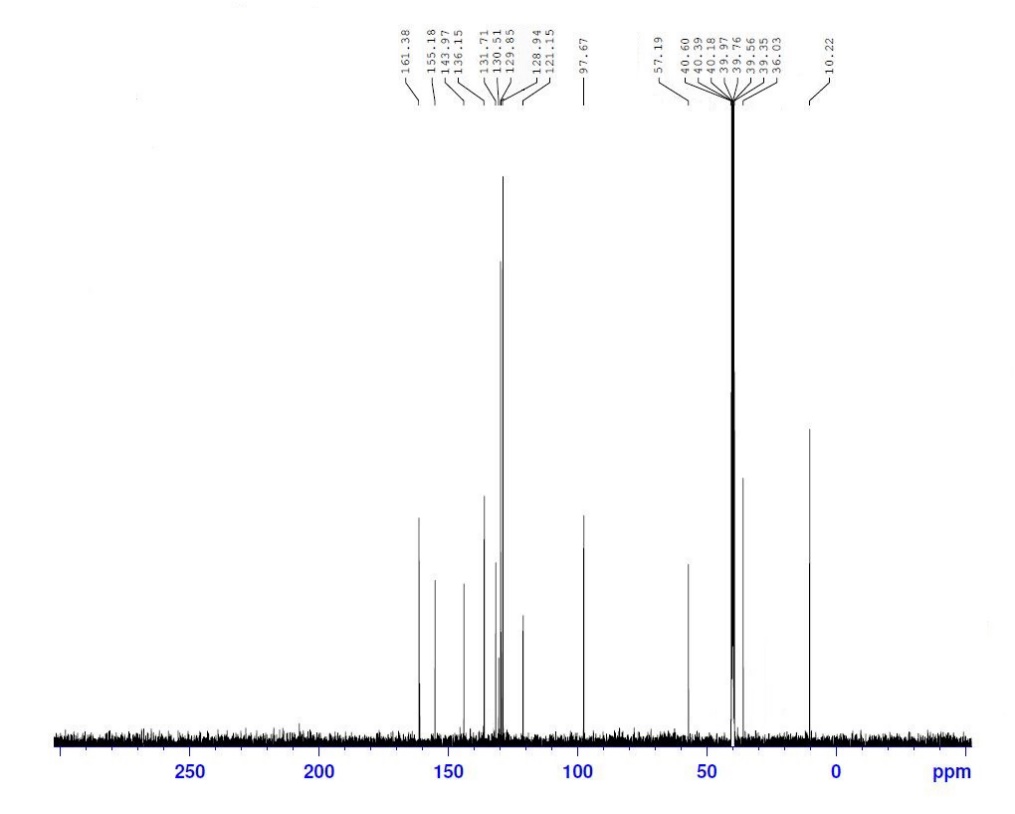


Fig. S6. The ^13^C NMR (100 MHz) spectrum of 6-Amino-4-(4-chlorophenyl)-3-methyl-1,4-dihydropyrano[2,3-*c*]pyrazole-5-carbonitrile

***6-Amino-3-methyl-4-(3-nitrophenyl)-1,4-dihydropyrano[2,3-c]pyrazole-5-carbonitrile (Table 2, entry 3)***: White solid. m. p. 210-211 °C. FT- IR (ATR)/ ῡ(cm^-1^): 3230, 3115, 2187, 1640, 1595, 1519, 1488, 1406, 1349, 732. ^1^H NMR (400 MHz, DMSO-d_6_)/δ (ppm): 1.82 (s, 3H), 4.89 (s, 1H), 7.08 (s, 2H), 7.64 -7.70 (m, 2H), 8.04 (s, 1H), 8.13-8.15 (d, *J* = 8 Hz, 1H), 12.23 (s, 1H).; ^13^C NMR (100 MHz, DMSO-d_6_)/δ ppm: 161.63, 155.17, 148.36, 147.32, 136.38, 134.88, 130.47, 122.33, 121.01, 97.15, 56.59, 36.11, 10.25.

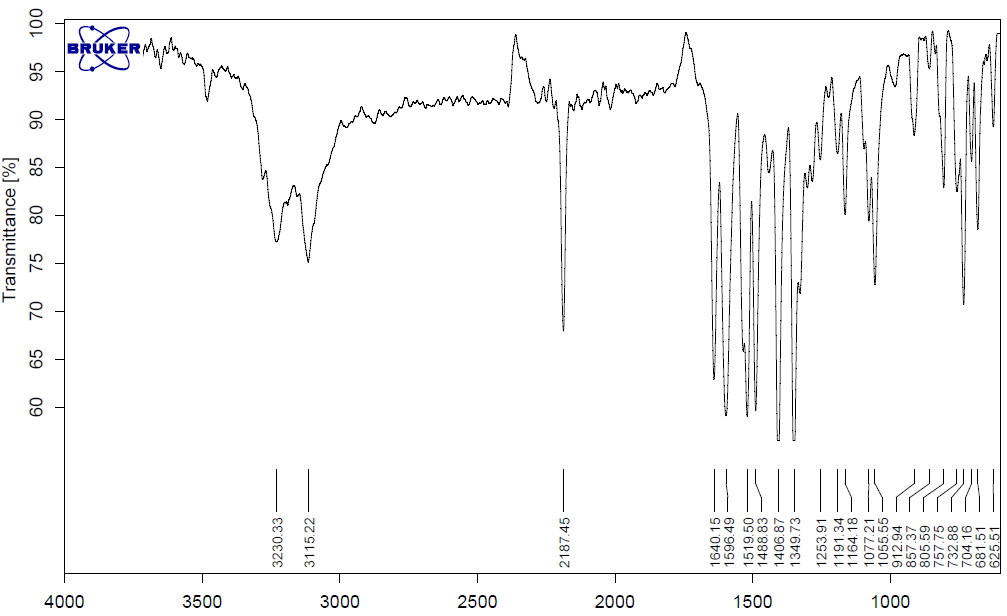


**Fig. S7.** The FT-IR of 6-Amino-3-methyl-4-(3-nitrophenyl)-1,4-dihydropyrano[2,3-*c*]pyrazole-5-carbonitrile


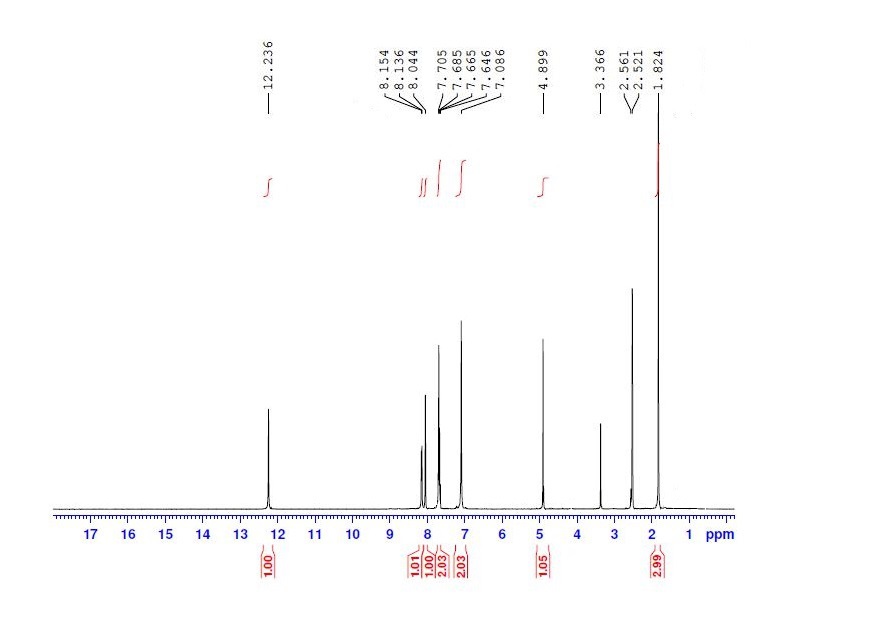


**Fig. S8.** The ^1^H NMR (400 MHz) spectrum of 6-Amino-3-methyl-4-(3-nitrophenyl)-1,4-dihydropyrano[2,3-*c*]pyrazole-5-carbonitrile


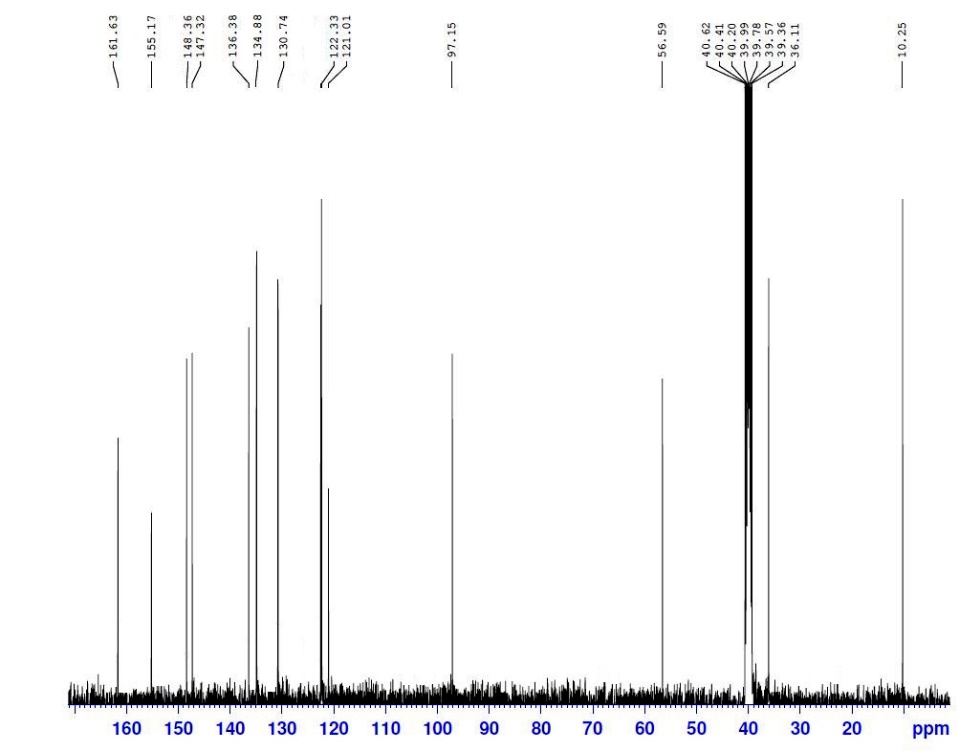


**Fig. S9.** The ^13^C NMR (100 MHz) spectrum of 6-Amino-3-methyl-4-(3-nitrophenyl)-1,4-dihydropyrano[2,3-*c*]pyrazole-5-carbonitrile

***6-Amino-4-(2,4-dichlorophenyl)-3-methyl-1,4-dihydropyrano[2,3-c]pyrazole-5-carbonitrile (Table 2, entry 4):*** Pale yellow solid. m. p. 223-225°C. FT- IR (ATR)/ ῡ(cm^-1^): 3394, 3295, 3120, 2177, 1649, 1591, 1494, 1409, 1098, 1050, 861, 750.; ^1^H NMR (400 MHz, DMSO-d_6_)/δ ppm: 1.85 (s, 3H), 5.13 (s, 1H), 7.07 (s, 2H), 7.29 (d, J = 8 Hz, 1H), 7.47 (dd, J = 8.4 Hz, J = 2 Hz, 1H), 7.65 (d, J = 2.4 Hz, 1H), 12.23 (s, 1H).; ^13^C NMR (100 MHz, DMSO-d_6_)/δ ppm: 161.30, 154.88, 140.07, 135.44, 132.81, 132.10, 128.83, 128.02, 120.25, 96.32, 55.21, 33.07, 9.53.


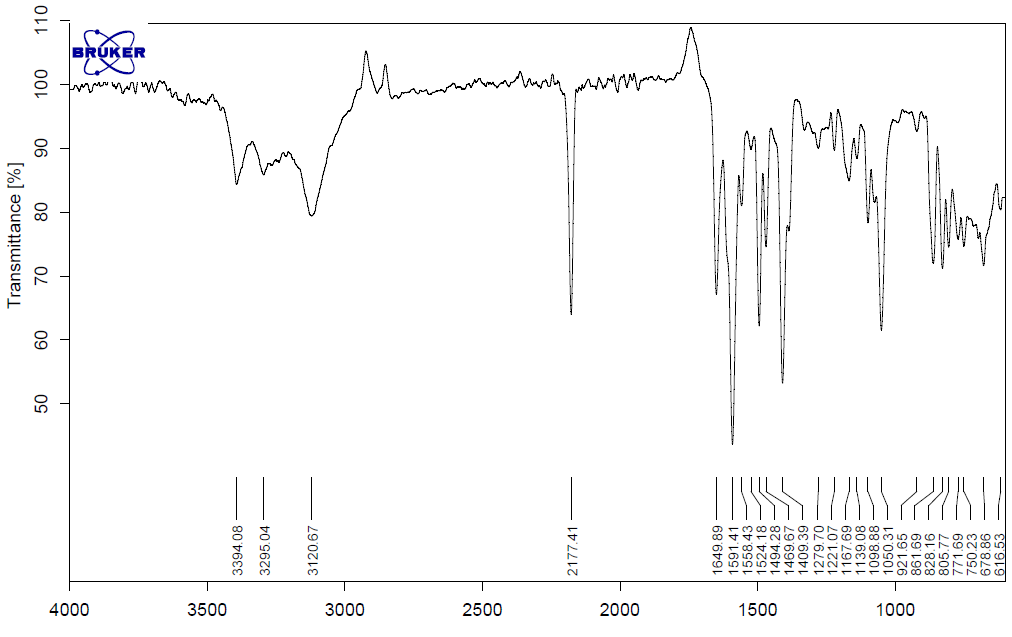


**Fig. S10.** The FT-IR of 6-Amino-4-(2,4-dichlorophenyl)-3-methyl-1,4-dihydropyrano[2,3-*c*]pyrazole-5-carbonitrile


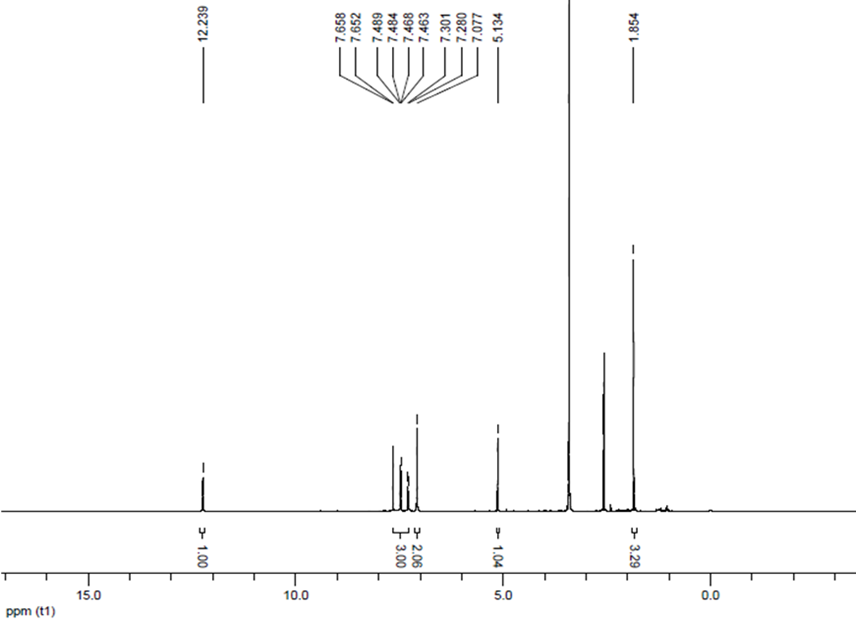


**Fig. S11.** The ^1^H NMR (400 MHz) spectrum of 6-Amino-4-(2,4-dichlorophenyl)-3-methyl-1,4-dihydropyrano[2,3-*c*]pyrazole-5-carbonitrile


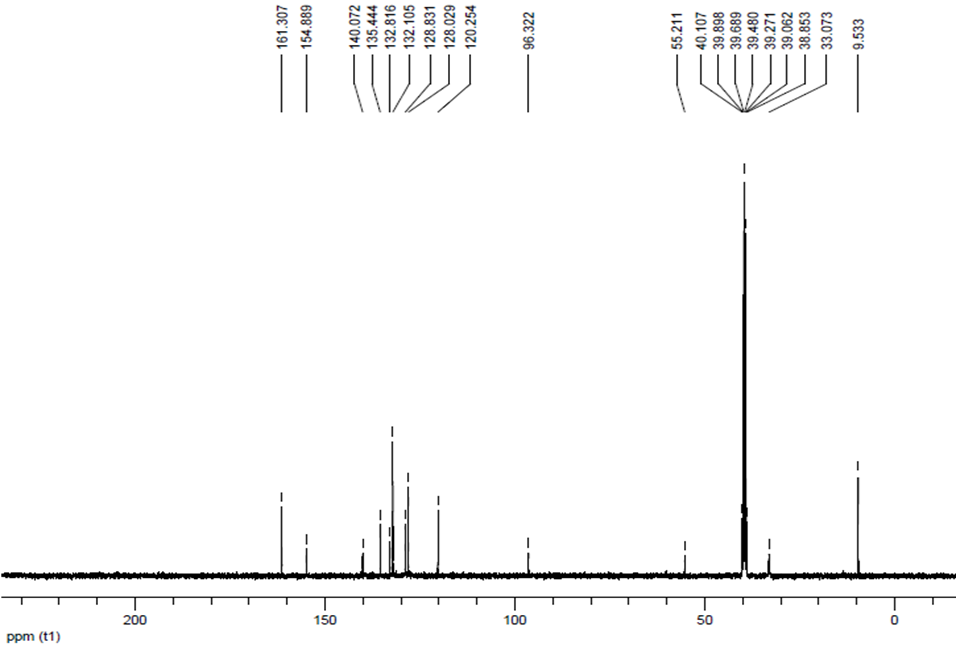


**Fig. S12.** The ^13^C NMR (100 MHz) spectrum of 6-Amino-4-(2,4-dichlorophenyl)-3-methyl-1,4-dihydropyrano[2,3-*c*]pyrazole-5-carbonitrile

***6-Amino-4-(4-fluorophenyl)-3-methyl-1,4-dihydropyrano[2,3-c]pyrazole-5-carbonitrile (Table 2, entry 5)***: White solid. m. p. 212-214 °C. FT- IR (ATR)/ ῡ (cm^-1^): 3476, 3228, 3117, 2194, 1638, 1596, 1505, 1404, 1224, 1051, 868. ^1^H NMR(400 MHz, DMSO-d_6_)/ δ ppm: 1.80 (s, 3H), 4.65 (s, 1H), 6.93 (s, 2H), 7.15 (t, *J* = 8 Hz, 2H), 7.20-7.24 (m, 2H), 12.14 (s, 1H).; ^13^C NMR (100 MHz, DMSO-d_6_)/δ ppm: 162.62, 161.30, 160.21, 155.17, 141.16, 136.10, 129.86, 121.20, 115.76, 97.97, 57.51, 35.90, 10.21.

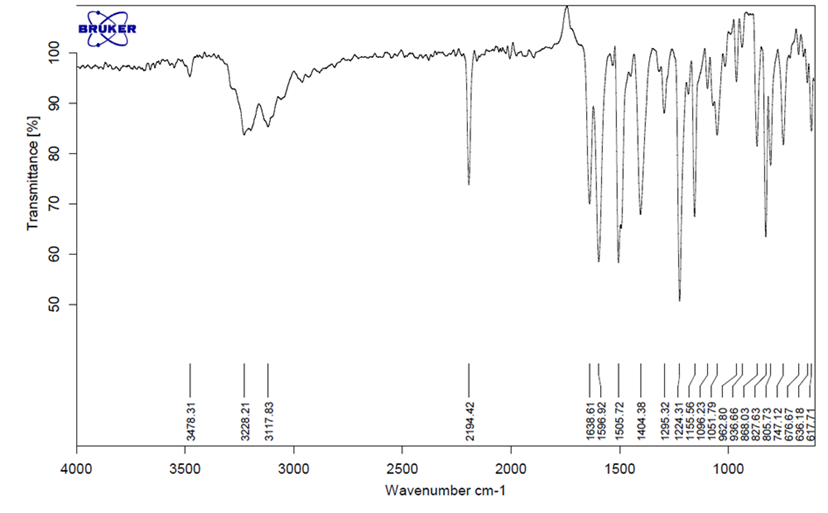


**Fig. S13.** The FT-IR of 6-Amino-4-(4-fluorophenyl)-3-methyl-1,4-dihydropyrano[2,3-*c*]pyrazole-5-carbonitrile


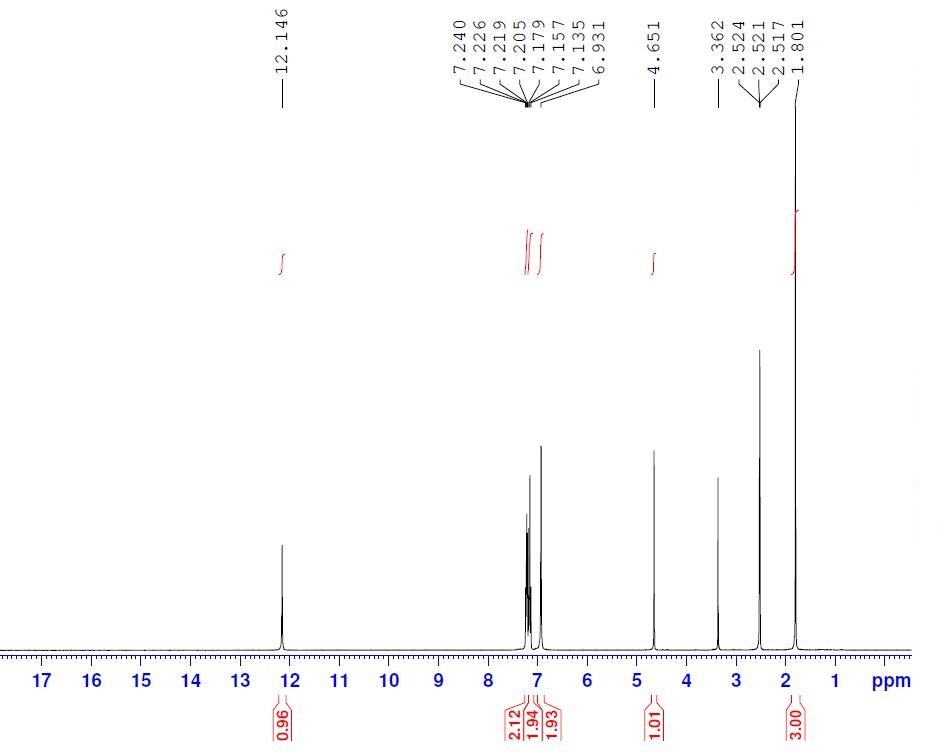


**Fig. S14.** The ^1^H NMR (400MHz) spectrum of 6-Amino-4-(4-fluorophenyl)-3-methyl-1,4-dihydropyrano[2,3-*c*]pyrazole-5-carbonitrile


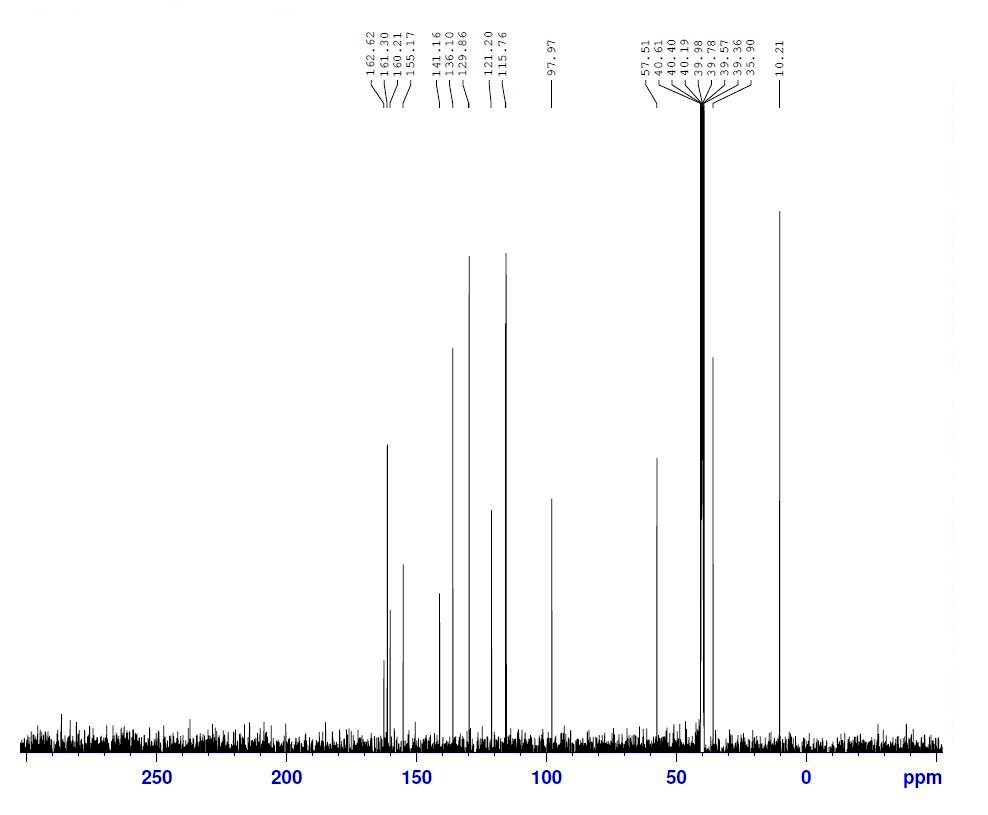


**Fig. S15.** The ^13^C NMR (100 MHz) spectrum of 6-Amino-4-(4-fluorophenyl)-3-methyl-1,4-dihydropyrano[2,3-*c*]pyrazole-5-carbonitrile

***6-Amino-4-(4-bromophenyl)-3-methyl-1,4-dihydropyrano[2,3-c]pyrazole-5-carbonitrile (Table 2, entry 6)*** Cream solid. m. p. 178-180 °C. FT- IR (ATR)/ ῡ(cm^-1^): 3393, 3358, 3181, 2189, 1640, 1598, 1487, 1399, 1278, 1046, 798. ^1^H-NMR(400 MHz, DMSO-d_6_)/ δ ppm: 1.81 (s, 3H), 4.64 (s, 1H), 6.96 (s, 2H), 7.16-7.14 (d, *J* = 8 Hz, 2H), 7.54-7.52 (d, *J* = 8 Hz, 2H), 12.16 (s, 1H).; ^13^C NMR (100 MHz, DMSO-d_6_)/δ ppm: 161.40, 155.18, 144.40, 136.16, 132.51, 131.86, 130.70, 121.16, 120.24, 97.60, 57.12, 36.10, 10.24.


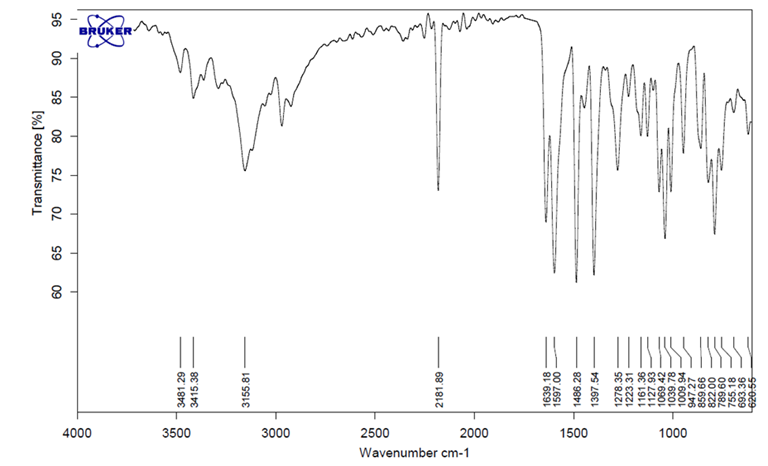


**Fig. S16.** The FT-IR of 6-Amino-4-(4-bromophenyl)-3-methyl-1,4-dihydropyrano[2,3-*c*]pyrazole-5-carbonitrile


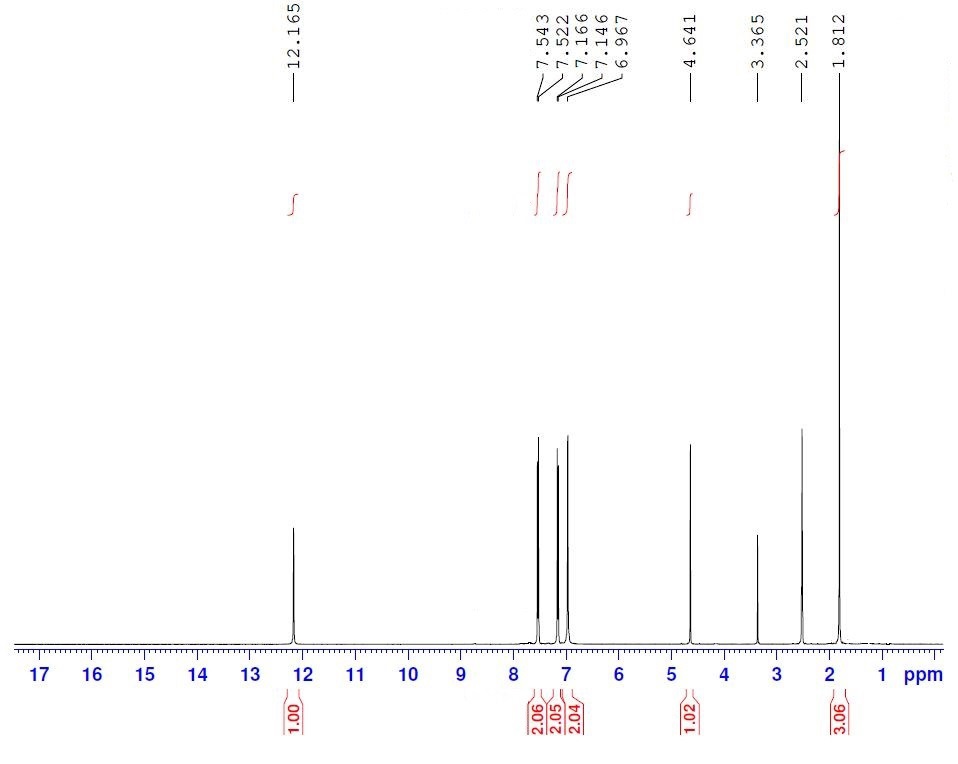


**Fig. S17.** The ^1^H NMR (400MHz) spectrum of 6-Amino-4-(4-bromophenyl)-3-methyl-1,4-dihydropyrano[2,3-*c*]pyrazole-5-carbonitrile


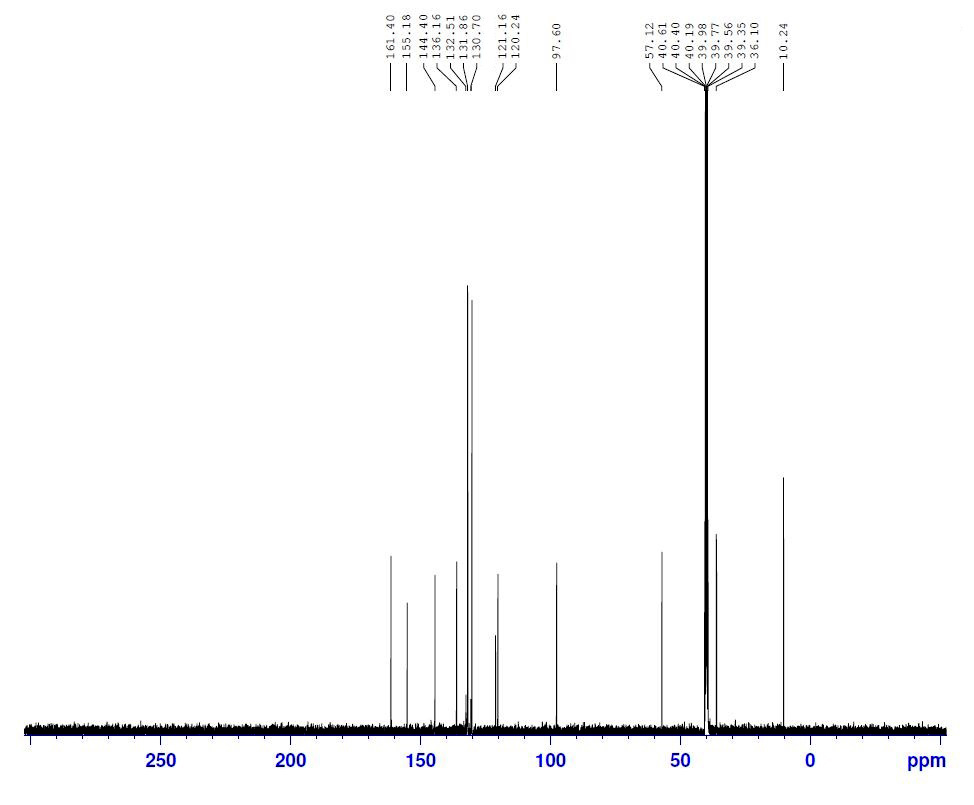


**Fig. S18.** The ^13^C NMR (100 MHz) spectrum of6-Amino-4-(4-bromophenyl)-3-methyl-1,4-dihydropyrano[2,3-*c*]pyrazole-5-carbonitrile

***6-Amino-4-(4-hydroxy-3-methoxyphenyl)-3-methyl-1,4-dihydropyrano[2,3-c]pyrazole-5-carbonitrile (Table 2, entry 7):*** Orange solid. m. p. 233-235 °C. FT-IR (ATR)/ ῡ(cm^-1^): 3488, 3406, 3325,3271, 3217, 2194, 1654, 1601, 1510, 1262, 1027, 743. ^1^H-NMR (400 MHz, DMSO-d_6_)/ δ ppm: 1.83 (s, 3H), 3.73 (s, 3H), 4.51 (s, 1H), 6.57 (s, 1H), 6.73-6.71 (d, *J* = 8 Hz, 2H), 6.83-6.88 (m, 2H), 9.73(s, 1H), 12.07 (s, 1H). ; ^13^C NMR (100 MHz, DMSO-d_6_)/δ ppm: 161.17, 155.19, 147.79, 145.70, 136.05, 123.99, 120.23, 115.94, 112.05, 110.42, 98.37, 58.04, 55.97, 36.31, 10.32.


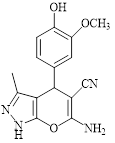


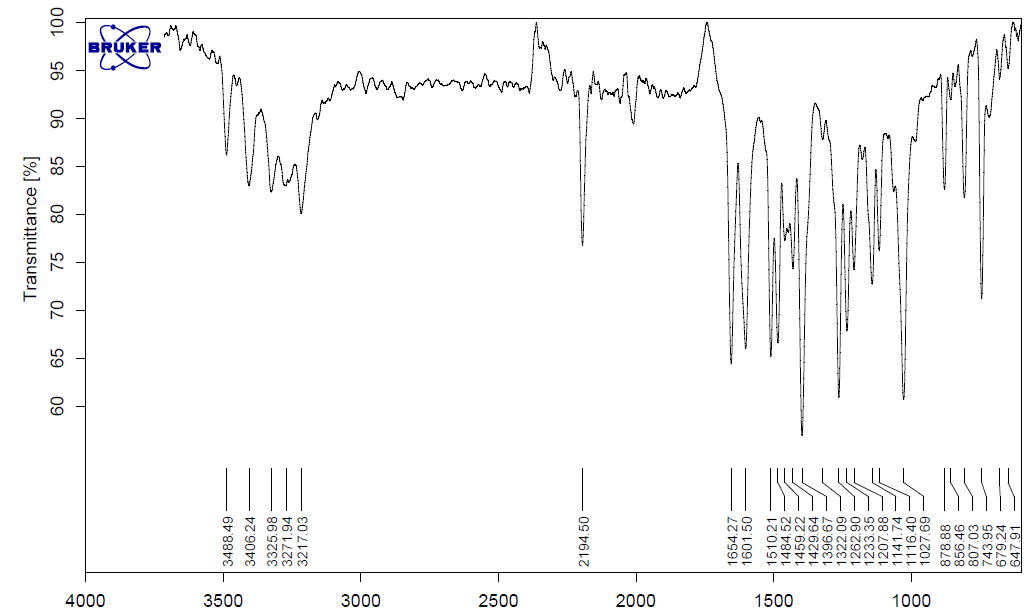


**Fig. S19.** The ^1^H NMR (400MHz) spectrum of 6-Amino-4-(4-hydroxy-3-methoxyphenyl)-3-methyl-1,4-dihydropyrano[2,3-c]pyrazole-5-carbonitrile


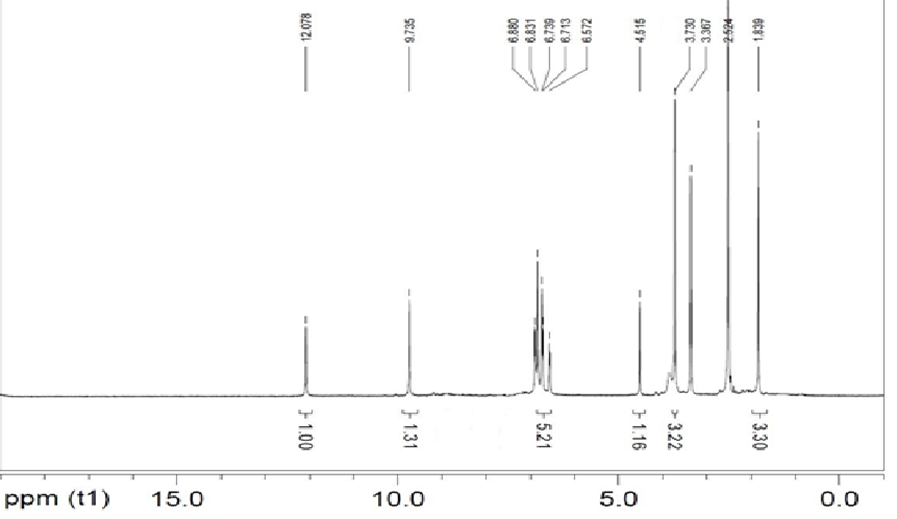


**Fig. S20.** The ^1^H NMR (400MHz) spectrum of 6-Amino-4-(4-hydroxy-3-methoxyphenyl)-3-methyl-1,4-dihydropyrano[2,3-c]pyrazole-5-carbonitrile

**
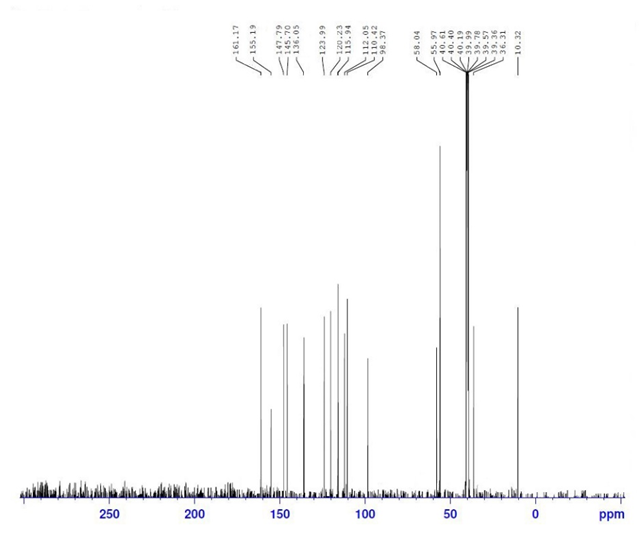
**

**Fig. S21.** The ^13^C NMR (100 MHz) spectrum of 6-Amino-4-(4-hydroxy-3-methoxyphenyl)-3-methyl-1,4-dihydropyrano[2,3-c]pyrazole-5-carbonitrile

***6-Amino-4-(4-hydroxyphenyl)-3-methyl-1,4-dihydropyrano[2,3-c]pyrazole-5-carbonitrile (Table 2, entry 8):*** Cream solid. m. p. 220-221 °C. FT- IR (ATR)/ ῡ(cm^-1^): 3367, 3134, 2175, 1646, 1596, 1512, 1491, 1406, 1190, 1044, 809. ^1^H NMR (250 MHz, Acetone-d_6_)/ δ(ppm): 1.74 (s, 3H), 4.44 (s, 1H), 6.65 (dd, *J*=7.5 Hz, *J*=3.7 Hz, 2H), 6.76 (br s, 2H), 6.91 (dd, *J*=7.5 Hz, *J*=3.7 Hz, 2H), 9.27 (s, 1H), 12.02 (s, 1H).; ^13^C NMR (100 MHz, DMSO-d_6_)/δ ppm: 161.10, 156.49, 155.22, 135.98, 135.24, 128.92, 121.40, 115.58, 98.54, 58.21, 35.95, 10.24.

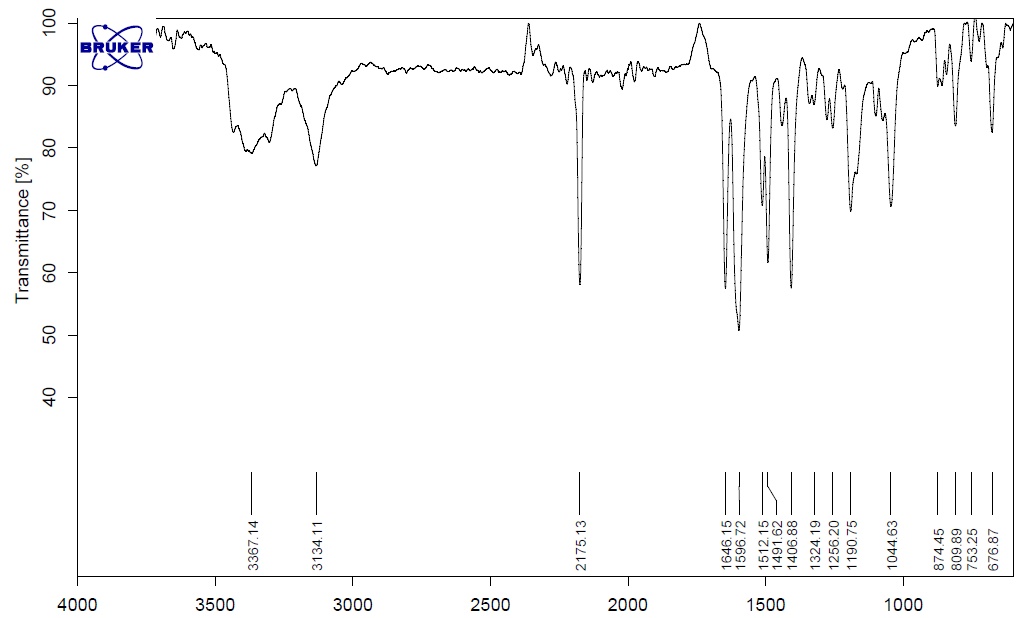


**Fig. S22.** The FT-IR of 6-Amino-4-(4-hydroxyphenyl)-3-methyl-1,4-dihydropyrano[2,3-*c*]pyrazole-5-carbonitrile


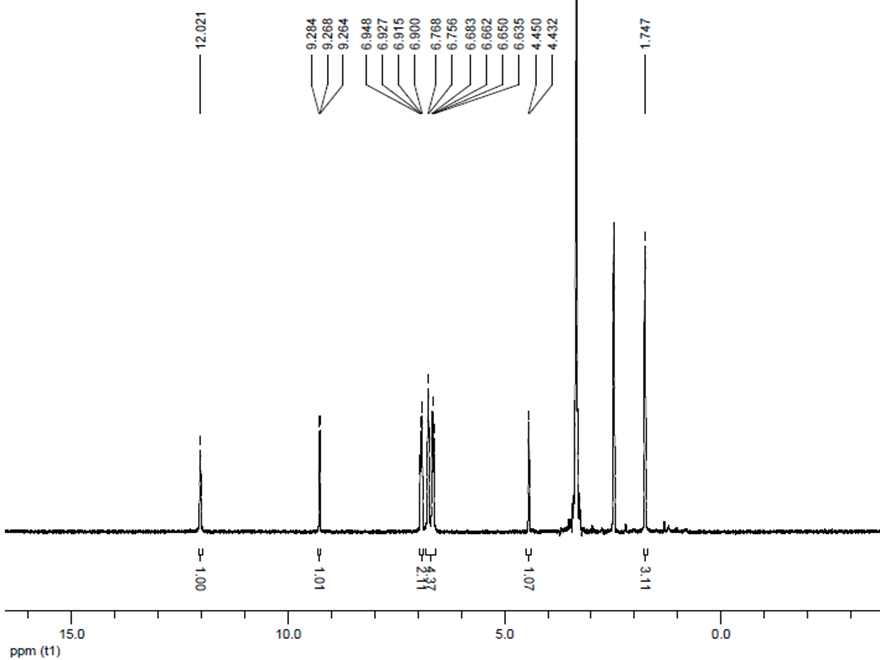


**Fig. S23.** The ^1^H NMR (250MHz) spectrum of 6-Amino-4-(4-hydroxyphenyl)-3-methyl-1,4-dihydropyrano[2,3-*c*]pyrazole-5-carbonitrile


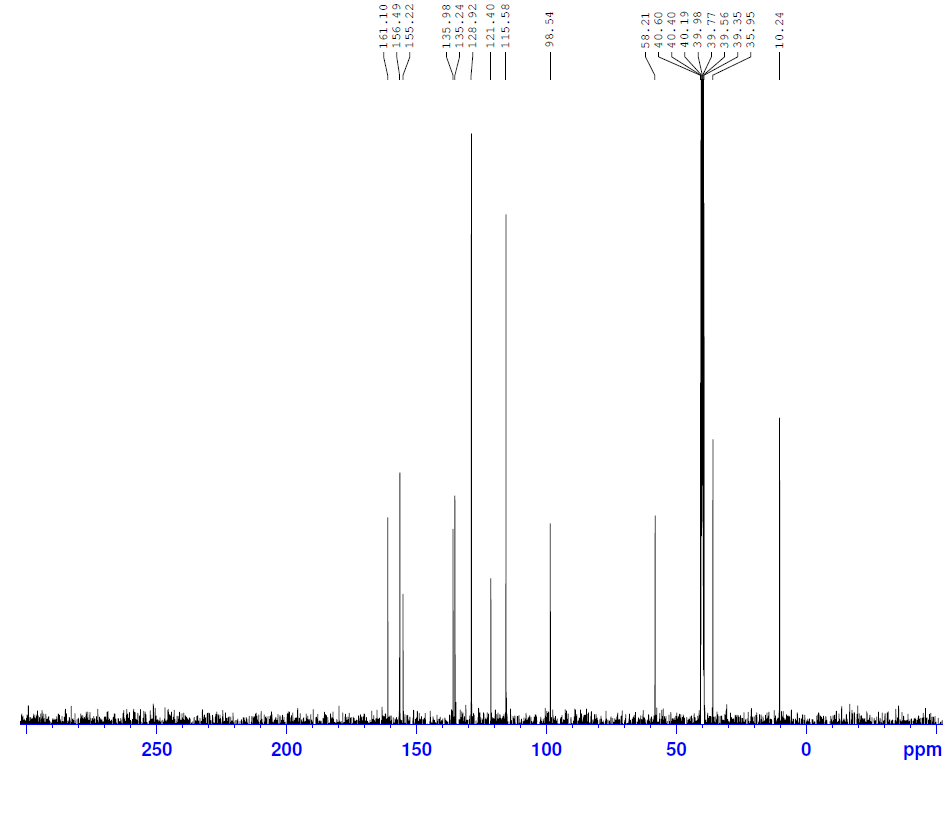


**Fig. S24.** The ^13^C NMR (100 MHz) spectrum of 6-Amino-4-(4-hydroxyphenyl)-3-methyl-1,4-dihydropyrano[2,3-*c*]pyrazole-5-carbonitrile

***6-Amino-3-methyl-4-(p-tolyl)-1,4-dihydropyrano[2,3-c]pyrazole-5-carbonitrile (Table 2, entry 9):*** Cream solid. m. p. 204-205 °C. FT- IR (ATR)/ ῡ(cm^-1^): 3360, 3171, 2182, 1649, 1598, 1490, 1401, 1046, 871.; ^1^H-NMR(400 MHz, DMSO-d_6_)/ δ ppm: 1.84 (s, 3H), 2.33 (s, 3H), 4.60 (s, 1H), 6.89 (s, 2H), 7.10 (d, *J* = 8 Hz, 2H), 7.17 (d, *J* = 8 Hz, 2H), 12.13 (s, 1H).


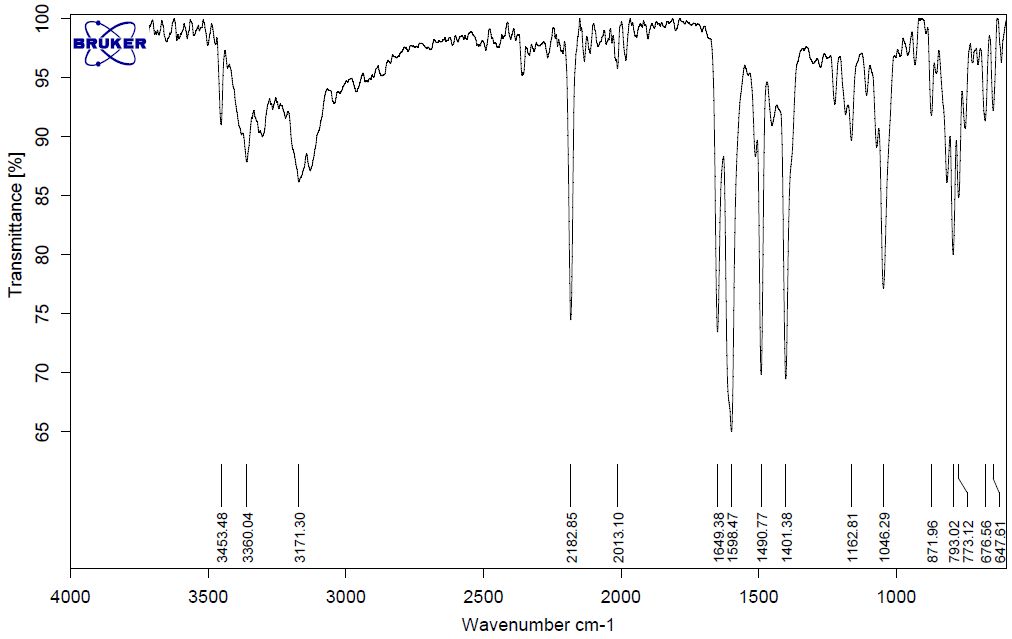


**Fig. S25.** The FT-IR of 6-Amino-3-methyl-4-(p-tolyl)-1,4-dihydropyrano[2,3-c]pyrazole-5-carbonitrile


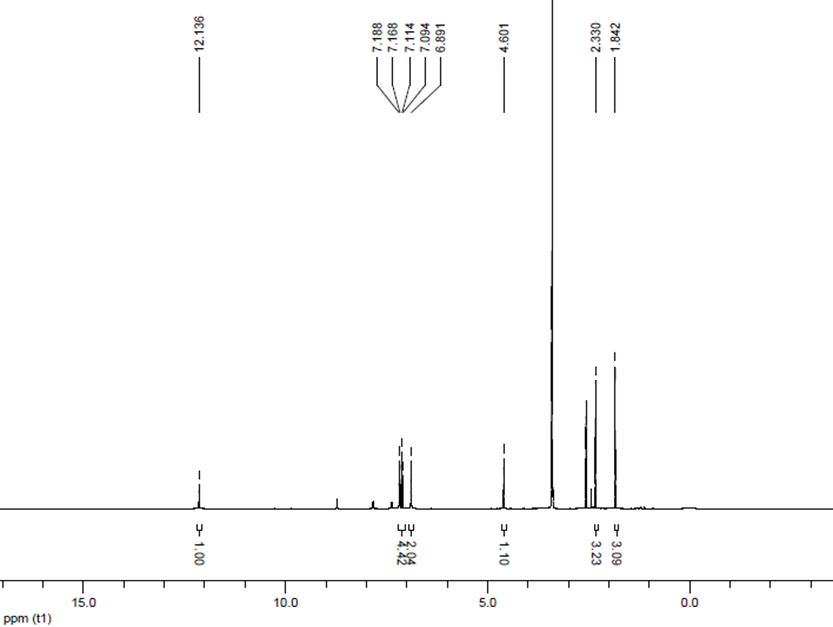


**Fig. S26.** The ^1^H NMR (400 MHz) spectrum of 6-Amino-3-methyl-4-(p-tolyl)-1,4-dihydropyrano[2,3-c]pyrazole-5-carbonitrile

*6-Amino-3-methyl-4-phenyl-1,4-dihydropyrano[2,3-c]pyrazole-5-carbonitrile (Table 2, entry 10):* White solid. m. p. 241-243 °C. FT- IR (ATR)/ ῡ (cm^-1^): 3371, 3176, 2191, 1647, 1596, 1488, 1044, 744.; ^1^HNMR (400 MHz, DMSO-d_6_)/δ (ppm): 1.86 (s, 3H), 4.67 (s, 1H), 6.95 (s, 2H), 7.25 (br s, 2H), 7.30 (br s, 1H), 7.40 (br s, 2H), 12.18 (s, 1H).

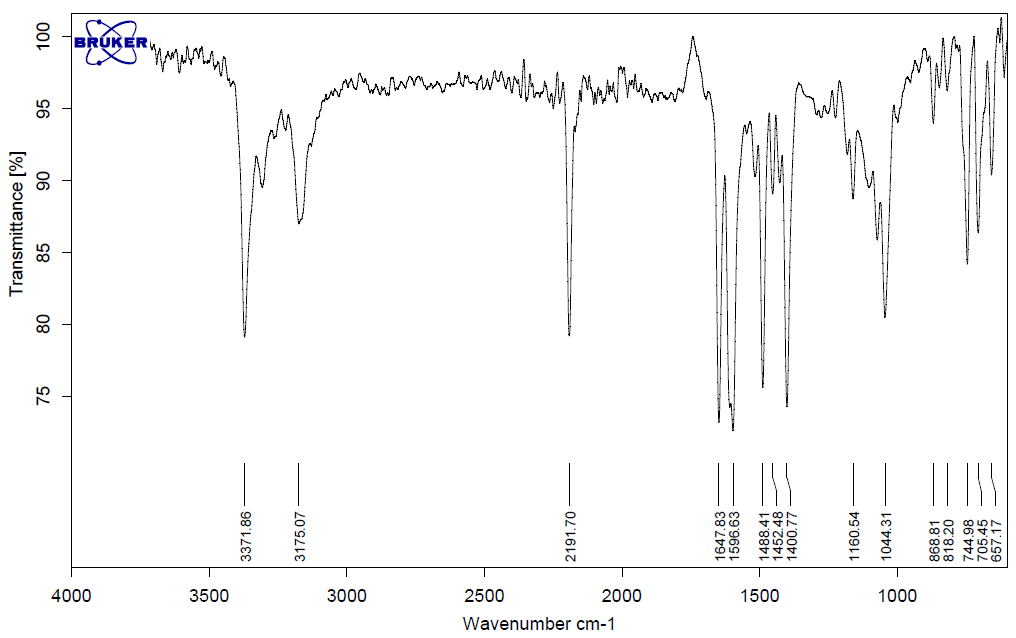


**Fig. S27.** The FT-IR of 6-Amino-3-methyl-4-phenyl-1,4-dihydropyrano[2,3-*c*]pyrazole-5-carbonitrile


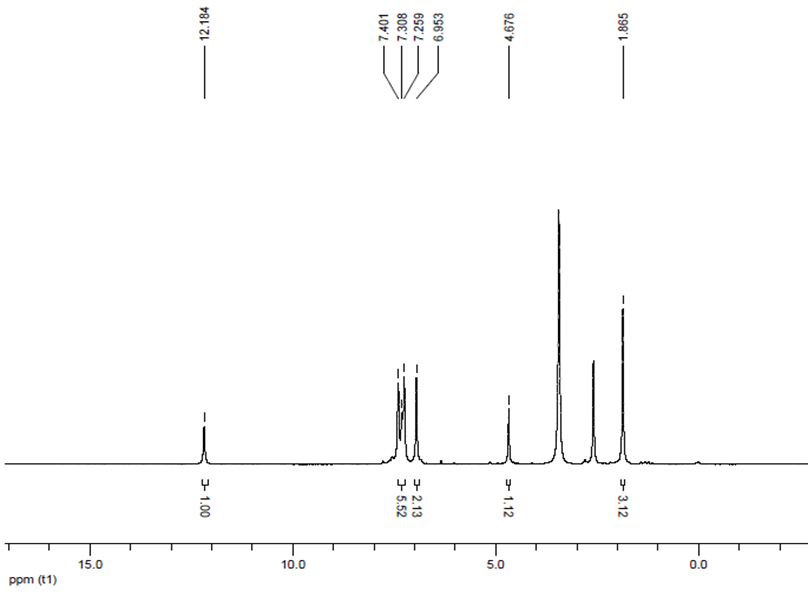


**Fig. S28.** The ^1^H NMR (400 MHz) spectrum of 6-Amino-3-methyl-4-phenyl-1,4-dihydropyrano[2,3-*c*]pyrazole-5-carbonitrile

***6-Amino-4-(3,4-dihydroxyphenyl)-3-methyl-1,4-dihydropyrano[2,3-c]pyrazole-5-carbonitrile (Table 2, entry 11)*** Brown solid. m. p. 221-224 °C. FT- IR (ATR)/ ῡ(cm^-1^): 3458, 3250, 3125, 2180, 1628, 1595, 1492, 1339, 1265, 1051, 755.; ^1^H NMR (250 MHz, DMSO-d_6_)/ δ ppm: 1.78 (s, 3H), 4.35 (s, 1H), 6.42-6.45 (m, 2H), 6.61 (dd, *J* = 7.5 Hz, *J* = 1.5 Hz, 1H), 6.75 (s, 2H), 8.70 (s, 1H), 8.82 (s, 1H), 12.02 (s, 1H). ^13^C NMR (100 MHz, DMSO-d_6_)/δ ppm: 161.06, 155.21, 145.67, 144.52, 136, 121.44, 118.75, 115.61, 115.04, 98.62, 58.31, 36.14, 10.26

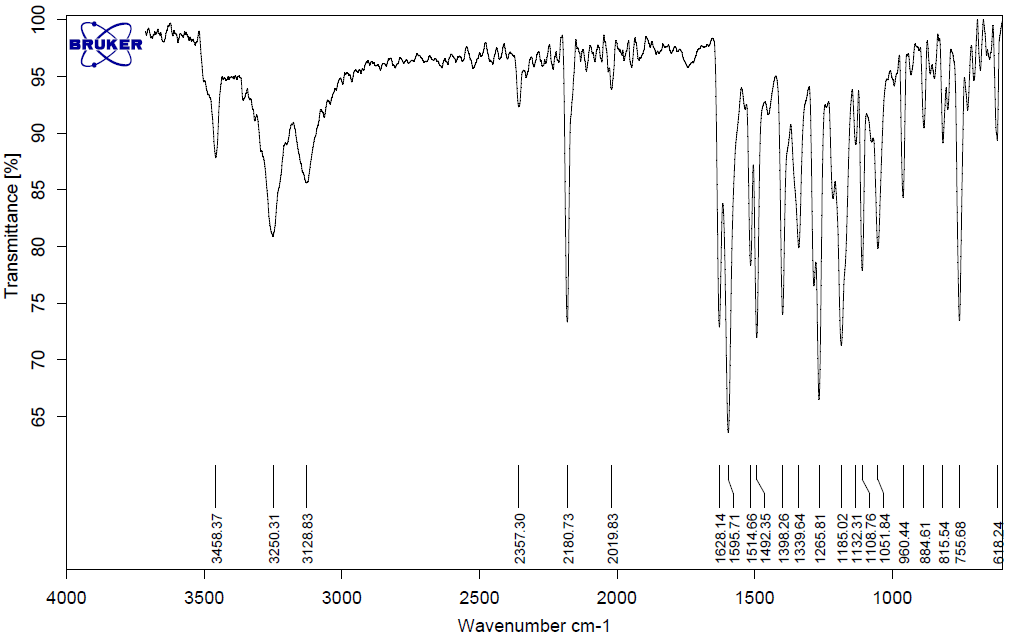


**Fig. S29.** The FT-IR of 6-Amino-4-(3,4-dihydroxyphenyl)-3-methyl-1,4-dihydropyrano[2,3-c]pyrazole-5-carbonitrile


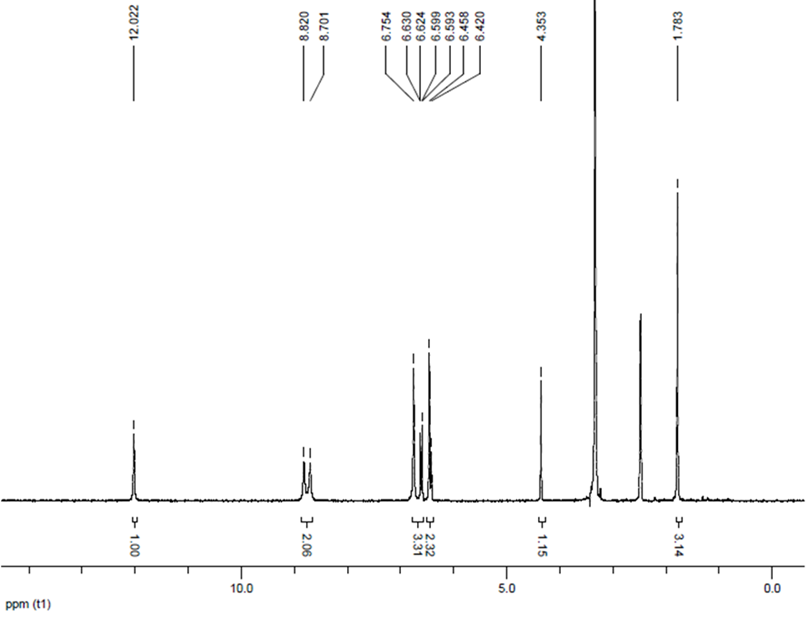


**Fig. S30.** The ^1^H NMR (250 MHz) spectrum of 6-Amino-4-(3,4-dihydroxyphenyl)-3-methyl-1,4-dihydropyrano[2,3-c]pyrazole-5-carbonitrile


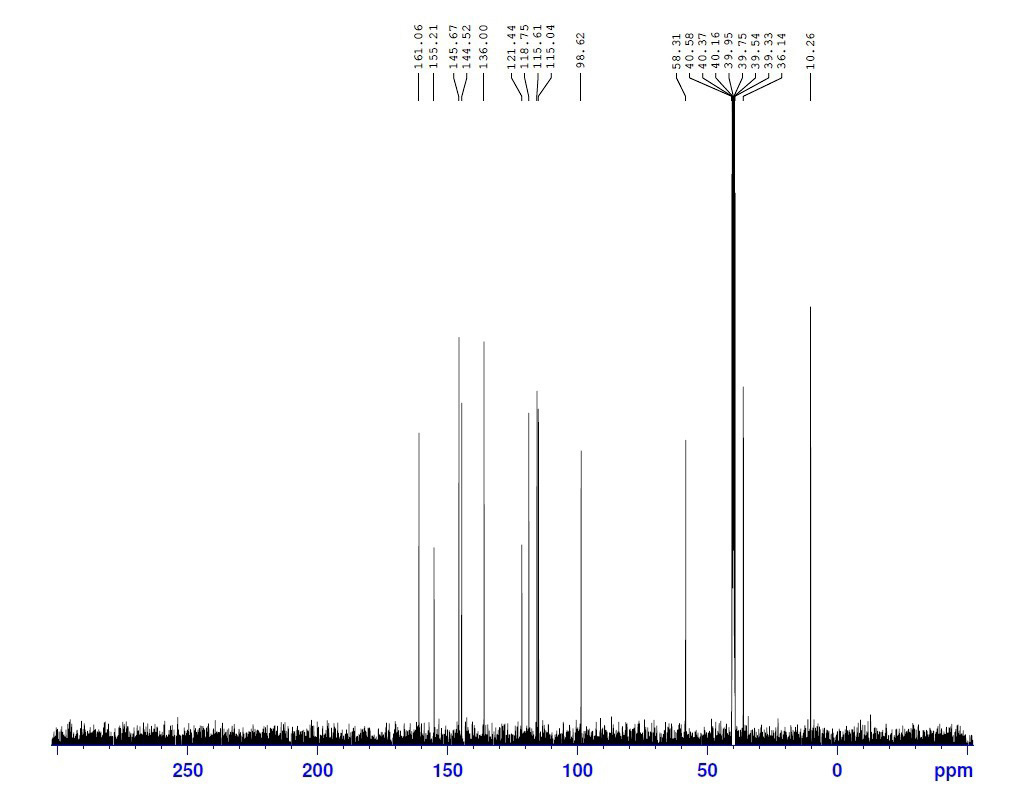


**Fig. S31.** The ^13^C NMR (100 MHz) spectrum of 6-Amino-4-(3,4-dihydroxyphenyl)-3-methyl-1,4-dihydropyrano[2,3-c]pyrazole-5-carbonitrile

*6-Amino-4-(furan-2-yl)-3-methyl-1,4-dihydropyrano[2,3-c]pyrazole-5-carbonitrile (Table 2, entry 12)*Cream solid. m. p. 234-235 °C. FT- IR (ATR) /ῡ (cm^-1)^ = 3357, 3179, 2190, 1645, 1598, 1492, 1405, 1152, 1048, 1009, 751, 656.; ^1^H NMR (250 MHz, Acetone-d_6_) δ/ ppm: 1.94 (s, 3H), 4.75 (s, 1H), 6.15-6.17 (m, 1H), 6.32-6.34 (m, 1H), 6.92 (br s, 2H), 7.49-7.51 (m, 1H), 12.14 (s, 1H). ^13^C NMR (100 MHz, DMSO-d_6_)/δ ppm: 161.96, 156.19, 155.29, 142.75, 136.31, 121.08, 110.72 106.12, 95.59, 54.43, 30.28, 10.05.

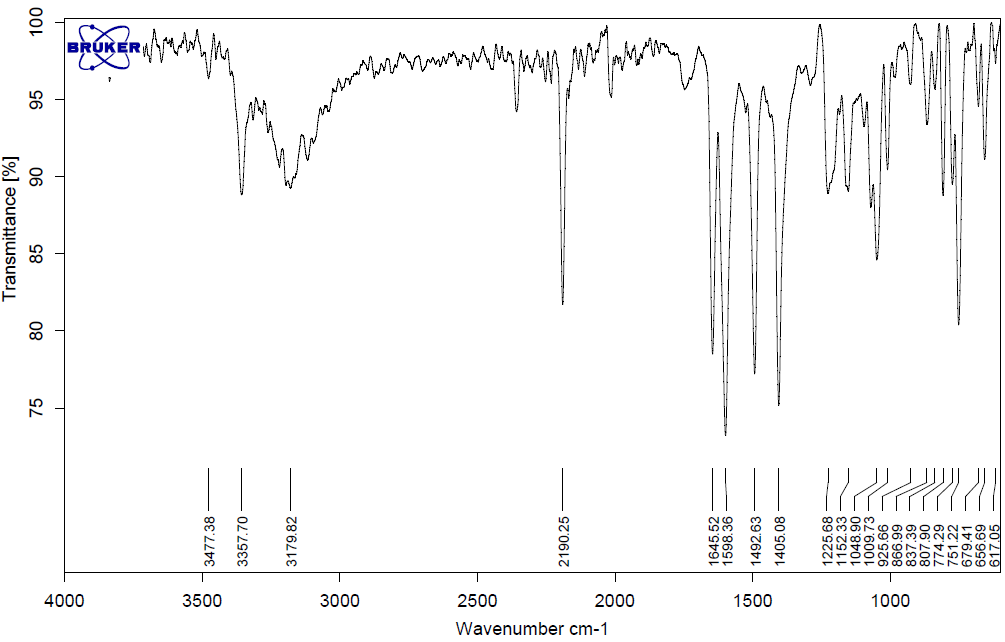


**Fig. S32.** The FT-IR of 6-Amino-4-(furan-2-yl)-3-methyl-1,4-dihydropyrano[2,3-*c*]pyrazole-5-carbonitrile


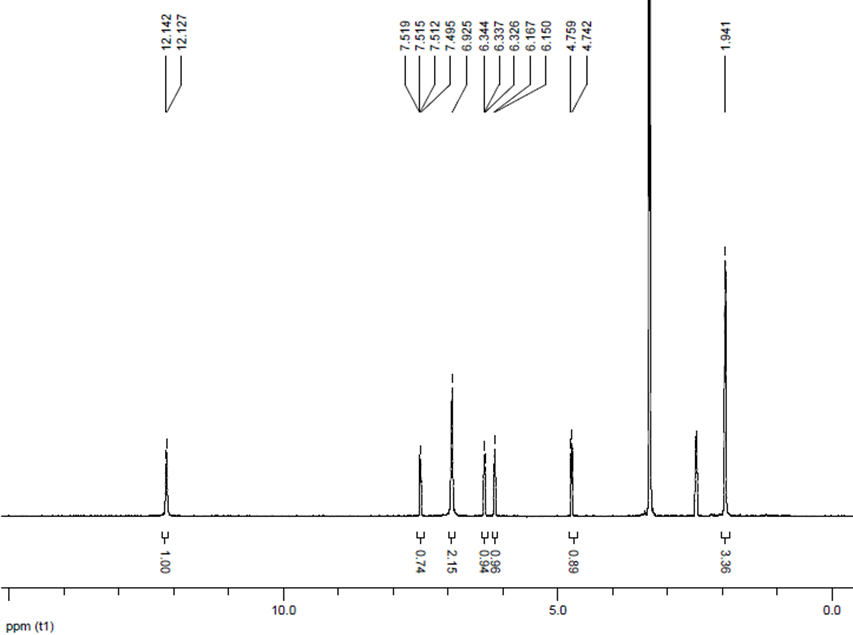


**Fig. S33.** The ^1^H NMR (250 MHz) spectrum of 6-Amino-4-(furan-2-yl)-3-methyl-1,4-dihydropyrano[2,3-*c*]pyrazole-5-carbonitrile


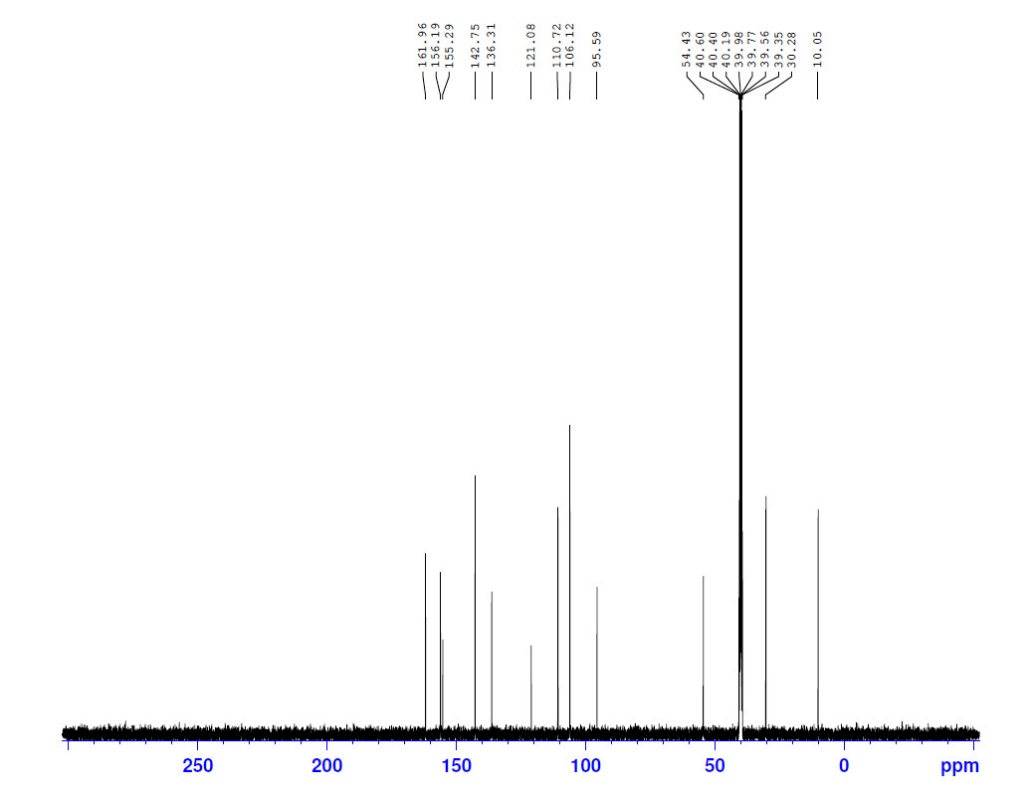


**Fig. S34.** The ^13^C NMR (100 MHz) spectrum of 6-Amino-4-(furan-2-yl)-3-methyl-1,4-dihydropyrano[2,3-*c*]pyrazole-5-carbonitrile

***6-Amino-3-methyl-4-(1H-pyrrol-2-yl)-1,4-dihydropyrano[2,3-c]pyrazole-5-carbonitrile (Table 2, entry 13):*** Brown solid, m. p. 210-213°C. FT-IR (ATR) ῡ (cm^-1^): 3308, 3181, 2191, 1638, 1593, 1489, 1402, 1034, 745. ^1^H NMR (400 MHz, DMSO-d_6_) /δ ppm: 12.04 (s, NH, 1H), 10.52 (s, NH, 1H), 6.78 (s, NH, 2H), 6.57 (d, *J*=1.6, 1H), 5.88-5.90 (m, 1H), 5.85 (br s, 1H), 4.64 (s, 1H), 1.84 (s, 3H). ^13^C NMR (100 MHz, DMSO-d_6_,) /δ ppm: 161.24, 155.16, 136.16, 133.98, 121.44, 117.61, 107.26, 105.62, 97.39, 57.03, 30.02, 9.98.

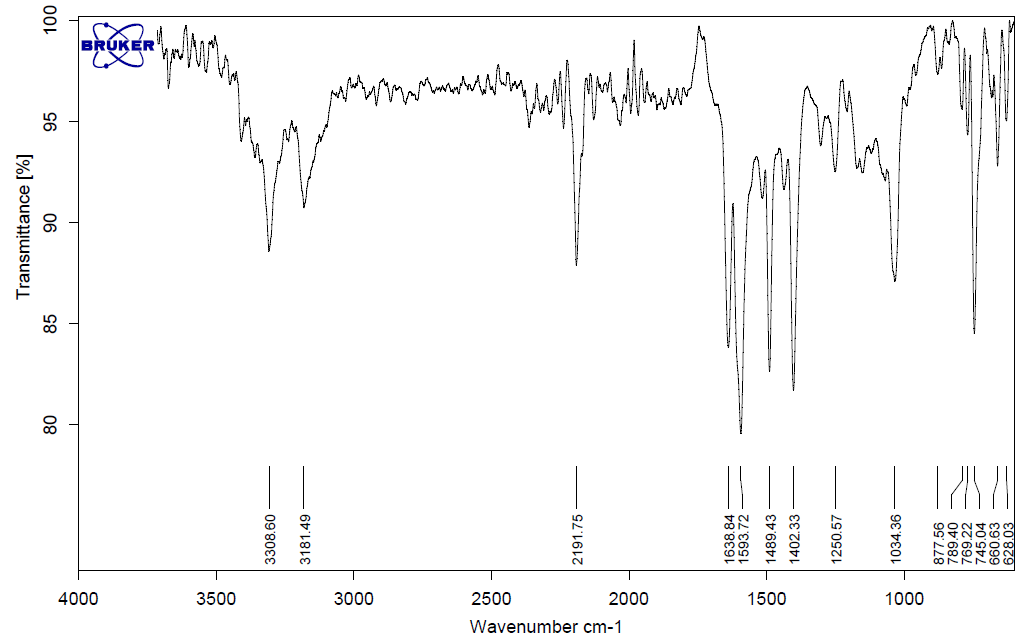


**Fig. S35.** The FT-IR of 6-Amino-3-methyl-4-(1H-pyrrol-2-yl)-1,4-dihydropyrano[2,3-c]pyrazole-5-carbonitrile


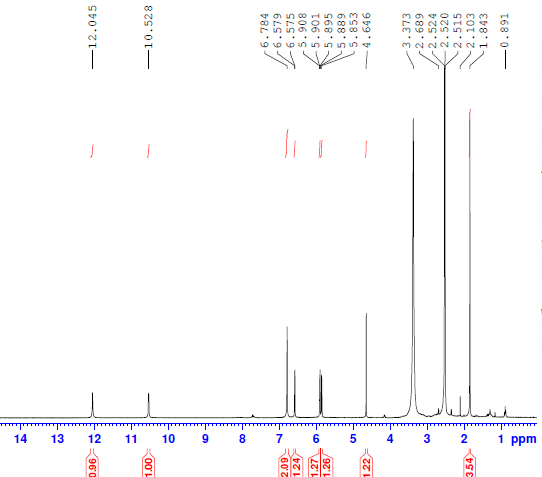


**Fig. S36.** The ^1^H NMR (400MHz) spectrum of 6-Amino-3-methyl-4-(1H-pyrrol-2-yl)-1,4-dihydropyrano[2,3-c]pyrazole-5-carbonitrile


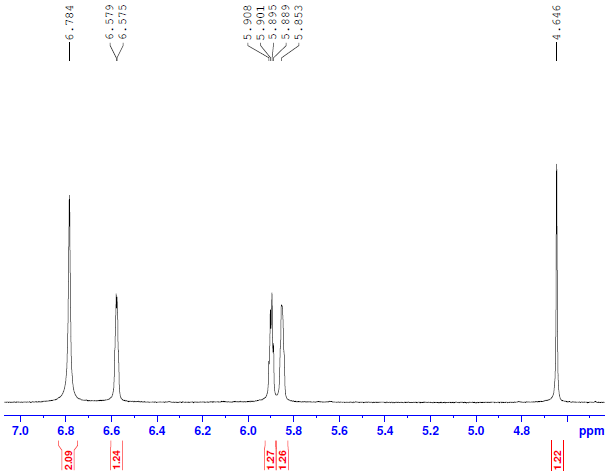


**Fig. S37.** The ^1^H NMR (400MHz) spectrum of 6-Amino-3-methyl-4-(1H-pyrrol-2-yl)-1,4-dihydropyrano[2,3-c]pyrazole-5-carbonitrile


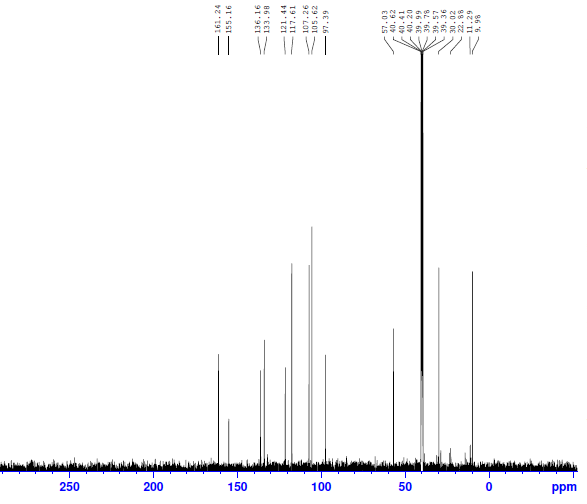


**Fig. S38.** The ^13^C NMR (100MHz) spectrum of 6-Amino-3-methyl-4-(1H-pyrrol-2-yl)-1,4-dihydropyrano[2,3-c]pyrazole-5-carbonitrile

***6-Amino-4-(2-methoxyphenyl)-3-methyl-1,4-dihydropyrano[2,3-c]pyrazole-5-carbonitrile (Table 2, entry 14)***Yellow solid. m. p. 220-222 °C. FT- IR (ATR)/ ῡ(cm^-1^ ): 3374, 3310, 3154, 2193, 1597, 1486, 1241, 1159, 1104, 1026, 762.; ^1^HNMR (400 MHz, DMSO-d_6_)/ δ ppm: 1.88 (s, 3H), 3.87 (s, 3H), 5.60 (s, 1H), 6.89 (s, 2H), 6.99 (t, *J* = 7.2, 1H), 7.06-7.10 (m, 2H), 7.27-7.31 (m, 1H), 12.09 (s, 1H).; ^13^C NMR (100 MHz, DMSO-d_6_)/δ ppm: 161.44, 156.30, 155.04, 134.99, 132.07, 128.58, 127.87, 120.86, 120.78, 111.25, 97.79, 56.30, 55.53, 29.09, 9.47.


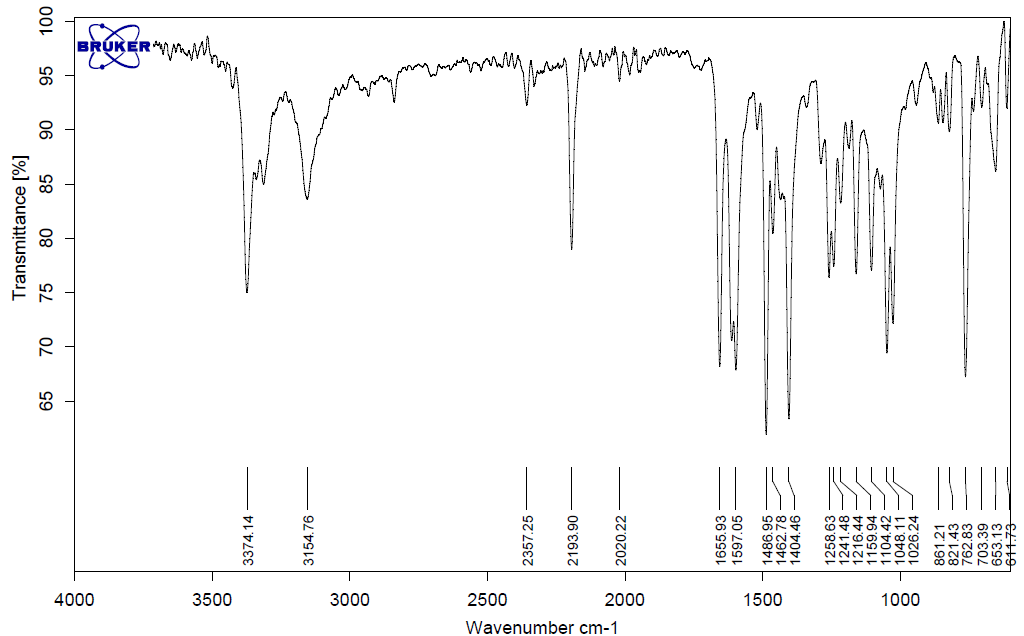


**Fig. S39.** The FT-IR of 6-Amino-4-(2-methoxyphenyl)-3-methyl-1,4-dihydropyrano[2,3-*c*]pyrazole-5-carbonitrile


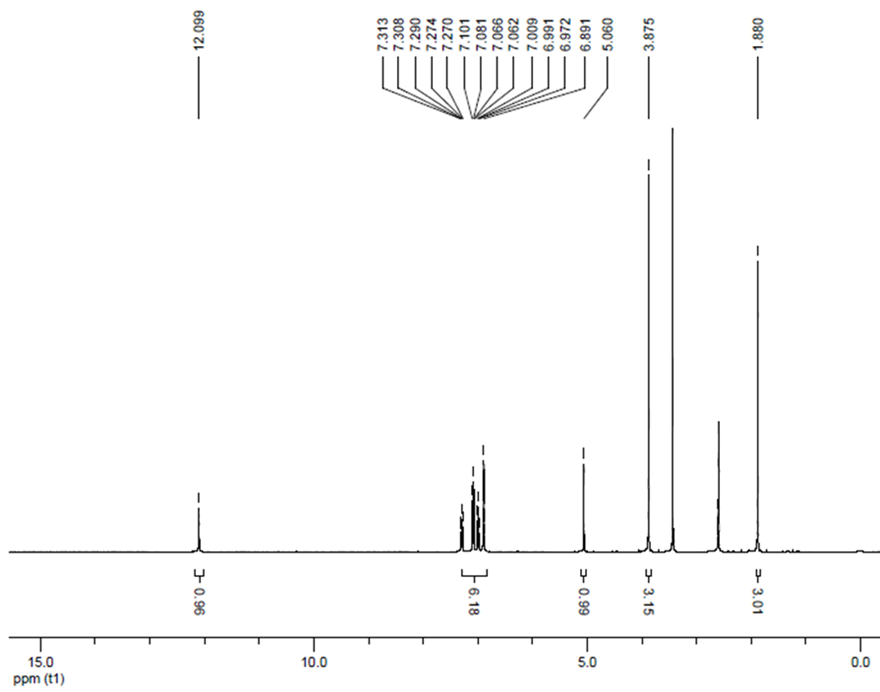


**Fig. S40.** The ^1^H NMR (400 MHz) spectrum of 6-Amino-4-(2-methoxyphenyl)-3-methyl-1,4-dihydropyrano[2,3-*c*]pyrazole-5-carbonitrile


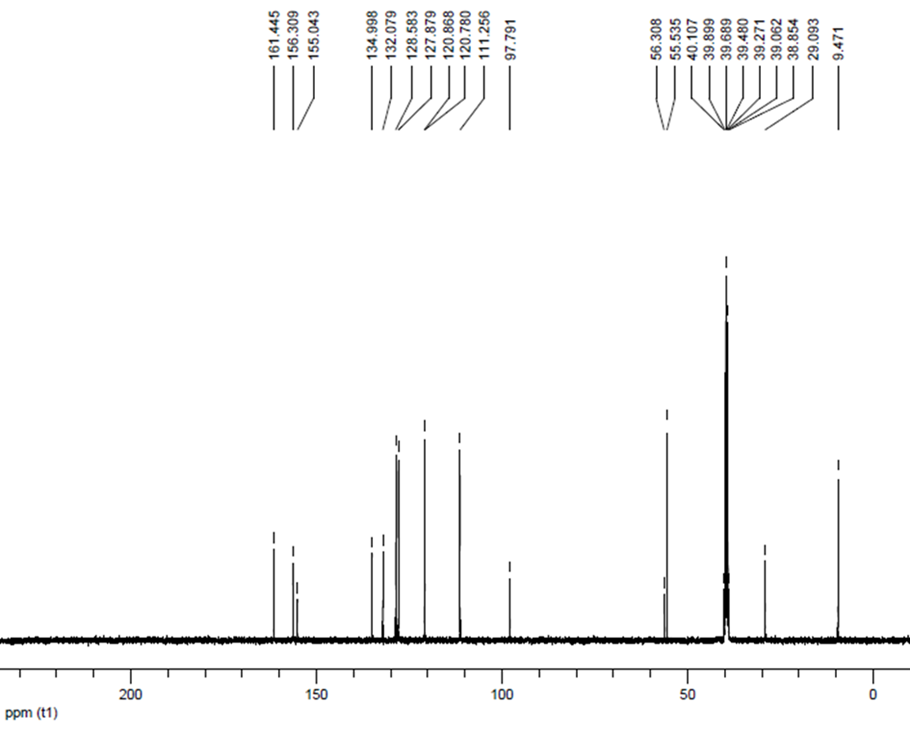


**Fig. S41.** The ^13^C NMR (100 MHz) spectrum of 6-Amino-4-(2-methoxyphenyl)-3-methyl-1,4-dihydropyrano[2,3-*c*]pyrazole-5-carbonitrile

***6-Amino-4-cyclohexyl-3-methyl-1,4-dihydropyrano[2,3-c]pyrazole-5-carbonitrile (Table 2 entry 15):*** White solid. m. p. 138-139 ºC FT- IR (ATR) ῡ (cm^-1)^:3462, 3293, 3165, 2925, 2852, 2173, 1637, 1584, 1491, 1065, 746 ^1^H NMR (500MHz, DMSO-*d_6_*)/ δ ppm: 0.86-1.67 (s, 12H), 2.15 (s, 3H), 6.79 (s, 2H), 12.03 (s, 1H).

**
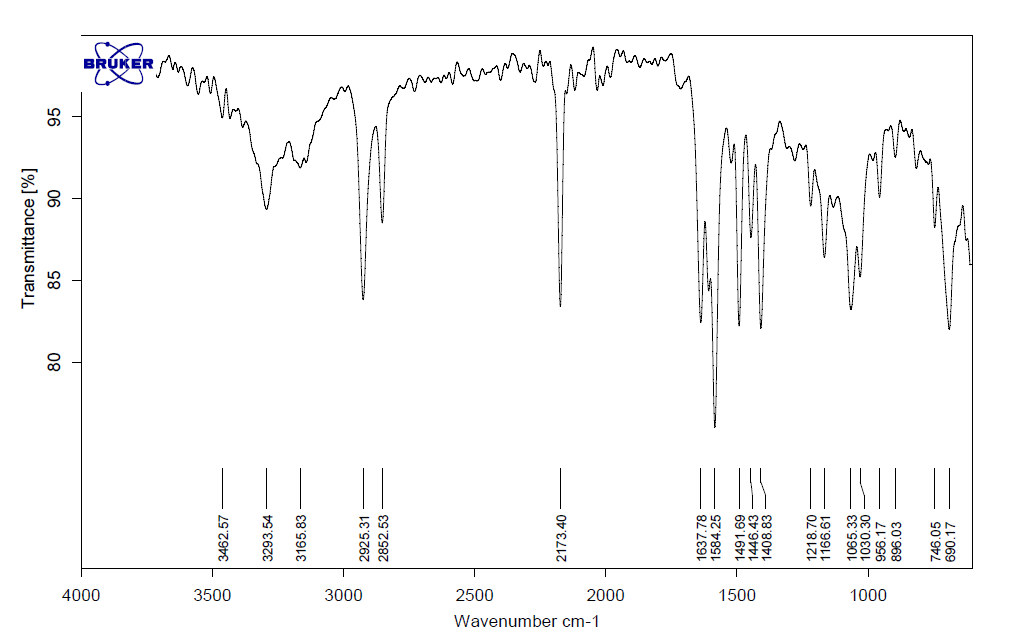
**

**Fig. S42.** The FT-IR spectrum of 6-Amino-4-cyclohexyl-3-methyl-1,4-dihydropyrano[2,3-*c*]pyrazole-5-carbonitrile.

**
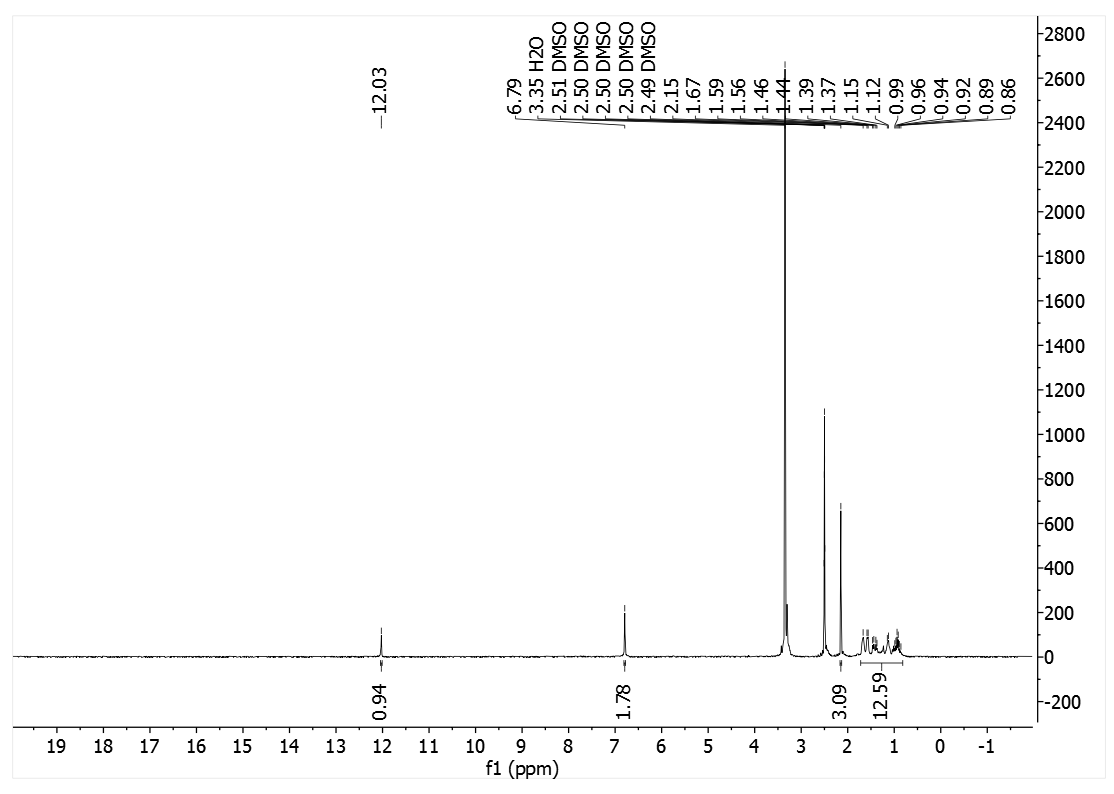
**

**Fig. S43.** The ^1^H NMR spectrum of 6-Amino-4-cyclohexyl-3-methyl-1,4-dihydropyrano[2,3-*c*]pyrazole-5-carbonitrile

***6-amino-3-methyl-4-phenyl-1,4-dihydropyrano[2,3-C]pyrazole-5-carbonitrile (Table 2 entry 16):*** White solid. m. p. 210-212 ºC FT- IR (ATR) ῡ (cm^-1)^:3334, 3061, 3026, 2926, 2858, 2174, 1603, 1495, 1061, 748.

**Fig. S44.** The FT-IR spectrum of 6-amino-3-methyl-4-phenyl-1,4-dihydropyrano[2,3-C]pyrazole-5-carbonitrile

***6-Amino-3-methyl-4-propyl-1,4-dihydropyrano[2,3-c]pyrazole-5-carbonitrile (Table 2 entry 17):*** White solid. m. p. 117-118 ºC. FT- IR (ATR) ῡ (cm^-1)^: 3347, 3169, 2991, 2932, 2203, 1695, 1596, 1496, 1071, 754. ^1^H NMR (500 MHz‎‏,‏‎ DMSO-*d_6_*)/ δ ppm‎‏:‏‎ 0.85-1.67(m, 11H), 6.89 (s, 2H), 12.12(s, 1H).

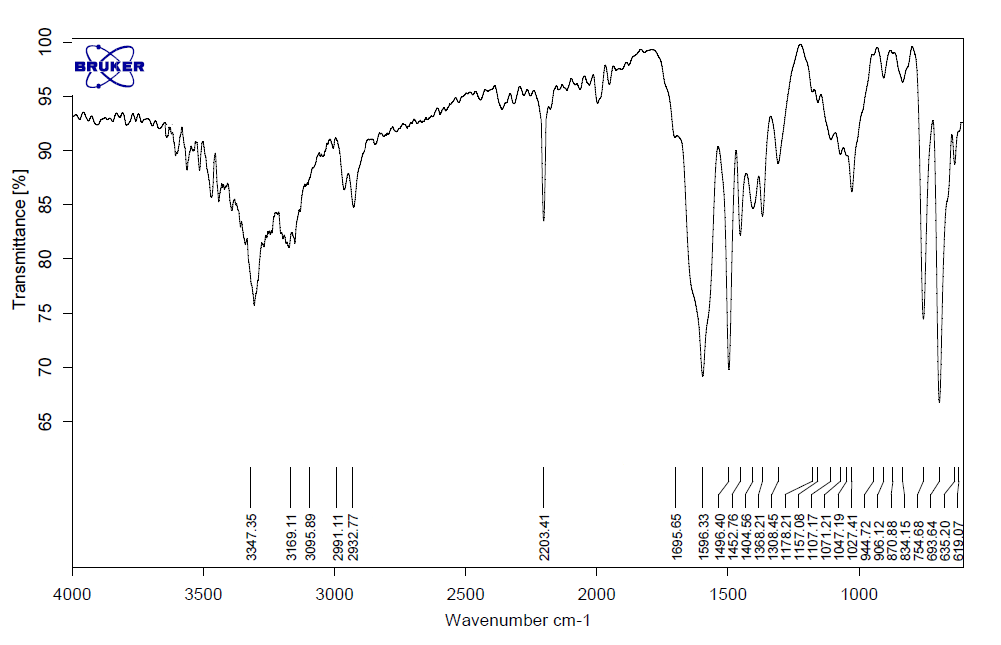


**Fig. S45.** The FT-IR spectrum of 6-Amino-3-methyl-4-propyl-1,4-dihydropyrano[2,3-*c*]pyrazole-5-carbonitrile


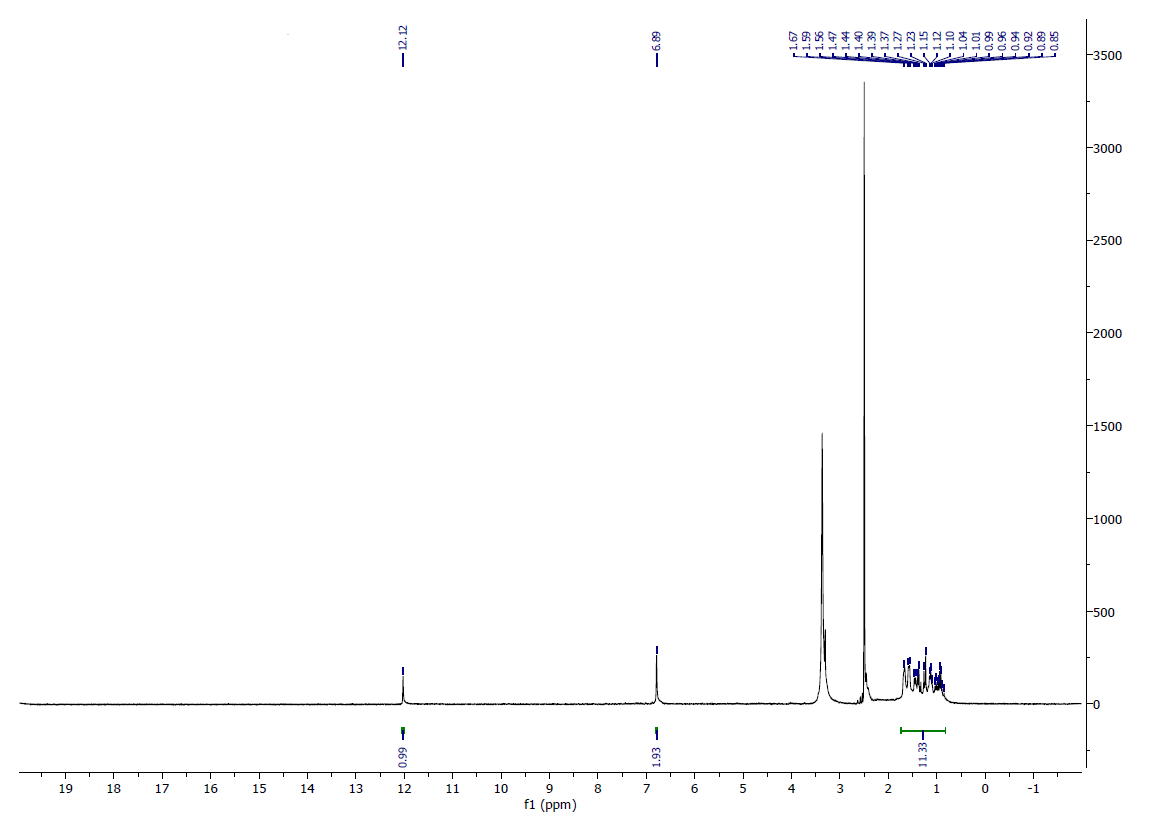


**Fig. S46.** The ^1^H NMR spectrum of 6-Amino-3-methyl-4-propyl-1,4-dihydropyrano[2,3-*c*]pyrazole-5-carbonitrile
